# Granulocytic myeloid-derived suppressor cell activity during biofilm infection is regulated by a glycolysis/HIF1a axis

**DOI:** 10.1172/JCI174051

**Published:** 2024-02-29

**Authors:** Christopher M. Horn, Prabhakar Arumugam, Zachary Van Roy, Cortney E. Heim, Rachel W. Fallet, Blake P. Bertrand, Dhananjay Shinde, Vinai C. Thomas, Svetlana G. Romanova, Tatiana K. Bronich, Curtis W. Hartman, Kevin L. Garvin, Tammy Kielian

**Affiliations:** 1 Department of Pathology, Microbiology, and Immunology and; 2 Department of Pharmaceutical Sciences, University of Nebraska Medical Center (UNMC), Omaha, Nebraska, USA.; 3 Department of Pharmacy, Northeastern University, Boston, Massachusetts, USA.; 4 Department of Orthopaedic Surgery and Rehabilitation, UNMC, Omaha, Nebraska, USA.

**Keywords:** Immunology, Infectious disease, Bacterial infections, Innate immunity, Orthopedics

## Abstract

*Staphylococcus aureus* is a leading cause of biofilm-associated prosthetic joint infection (PJI). A primary contributor to infection chronicity is an expansion of granulocytic myeloid-derived suppressor cells (G-MDSCs), which are critical for orchestrating the antiinflammatory biofilm milieu. Single-cell sequencing and bioinformatic metabolic algorithms were used to explore the link between G-MDSC metabolism and *S*. *aureus* PJI outcome. Glycolysis and the hypoxia response through HIF1a were significantly enriched in G-MDSCs. Interfering with both pathways in vivo, using a 2-deoxyglucose nanopreparation and granulocyte-targeted *Hif1a* conditional KO mice, respectively, attenuated G-MDSC–mediated immunosuppression and reduced bacterial burden in a mouse model of *S*. *aureus* PJI. In addition, single-cell RNA–Seq (scRNA-Seq) analysis of granulocytes from PJI patients also showed an enrichment in glycolysis and hypoxia-response genes. These findings support the importance of a glycolysis/HIF1a axis in promoting G-MDSC antiinflammatory activity and biofilm persistence during PJI.

## Abstract 2

A glycolysis/HIF1a axis promotes anti-inflammatory activity and biofilm persistence in granulocytic myeloid-derived suppressor cells during prosthetic joint infection.

## Graphical abstract

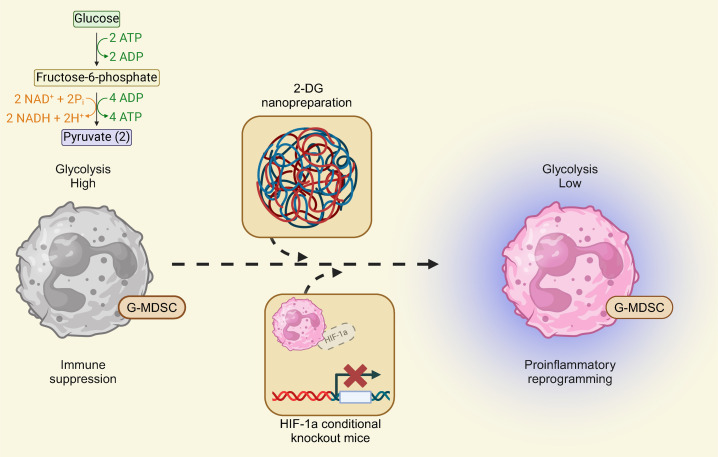

## Introduction

*Staphylococcus aureus* is a leading cause of healthcare- and community-associated infections (1). This is due, in part, to numerous virulence factors that allow the bacterium to adapt and thrive in a variety of host tissues (2, 3). The clinical management of *S*. *aureus* infections is further complicated by the rise in intrinsic antibiotic resistance and propensity to form biofilm. Biofilms are complex bacterial communities that secrete extracellular polymeric substances to promote aggregation and adherence to both biotic and abiotic surfaces (4, 5). Biofilm formation significantly increases infection chronicity due to the emergence of heterogenous zones within the biofilm that are limited in nutrient and oxygen availability (6, 7). These gradients lead to dramatic differences in core bacterial processes, such as metabolism, between the interior and exterior aspects of the biofilm (6). Collectively, these characteristics typically render antibiotic therapy alone ineffective (8, 9), which contributes to the difficulty in managing biofilm-associated prosthetic joint infections (PJIs), namely those that occur following total knee arthroplasty (TKA) and total hip arthroplasty (THA). PJI is a leading cause of early joint failure in patients who undergo primary and revisional TKA, where *S*. *aureus* is among the most frequently isolated pathogens (10). Currently, the most widely used approach for PJI treatment consists of a 2-stage revisional arthroplasty combined with long-term systemic antibiotics (11). Although this regimen is effective in most cases, the failure rate is still appreciably high (10%–20%) (12). With the steady rise in TKA and THA procedures, the healthcare complications originating from PJIs will continue to increase with time (13, 14).

Several studies have shown that *S*. *aureus* biofilms skew the host immune response toward an antiinflammatory phenotype that is beneficial for bacterial survival (15–20). Our laboratory has demonstrated that *S*. *aureus* biofilm infections, both in a mouse model of PJI and in humans, are characterized by a large influx of granulocytic myeloid-derived suppressor cells (G-MDSCs) (17, 19, 21–24). G-MDSCs are pathologically activated polymorphonuclear cells (PMNs) that are defined by their ability to suppress the proinflammatory activity of other immune cells, including T cells, macrophages, and PMNs (25). Although the mechanisms responsible for G-MDSC recruitment and expansion during *S*. *aureus* biofilm infection remain unclear, we have shown that their presence is critical for bacterial persistence during PJI (17, 23, 24). Furthermore, G-MDSCs are a primary source of IL-10, which mediates, in part, their immunosuppressive activity on other infiltrating leukocytes (17). We recently reported that biofilm-derived lactate is responsible for eliciting *Il10* expression in G-MDSCs by modulating histone deacetylase (HDAC) activity, highlighting the importance of bacterial-leukocyte metabolic crosstalk during *S*. *aureus* PJI (19).

Immunometabolism refers to links between specific metabolic programs (i.e., glycolysis, fatty acid oxidation) and immune cell function (i.e., pro-/antiinflammatory activity) (26–28). Regarding innate immunity, most work in this field has been performed with macrophages that increase their glycolytic flux to bolster anabolic processes and ROS production through the pentose phosphate pathway (PPP) in response to proinflammatory stimuli (26, 29, 30). This shift is accompanied by 2 breaks in the TCA cycle that serve to augment glycolysis by dampening TCA cycle activity and generating ROS by reverse electron flow (31, 32). Conversely, antiinflammatory macrophages increase TCA cycle activity to enhance oxidative metabolism (29, 33). Prior studies have indicated that PMNs rely solely on glycolysis under homeostatic conditions and a combination of glycolysis and the PPP during activation, which has been explained by their low mitochondrial abundance (34). However, recent work has revealed that PMNs can utilize alternative metabolic pathways to fuel their responses, including glycogenolysis and gluconeogenesis (35, 36). Although G-MDSCs are critical for *S*. *aureus* biofilm persistence (17, 19, 24), comparatively less is known about their metabolism or how it is influenced by biofilm formation in the context of PJI.

This study leveraged longitudinal single-cell RNA–Seq (scRNA-Seq) data collected from a mouse model of *S*. *aureus* PJI, which identified the enrichment of glycolysis and hypoxia signatures in G-MDSCs, which was confirmed with G-MDSC–biofilm cocultures in vitro. A critical role for G-MDSC glycolysis in vivo was demonstrated by the local delivery of a 2-deoxyglucose (2-DG) nanopreparation (NP) at the site of PJI that was preferentially internalized by G-MDSCs and impaired their antiinflammatory activity, resulting in improved biofilm clearance. Likewise, targeted *Hif1a* deletion in granulocytes decreased bacterial burden concomitant with transcriptional skewing of granulocyte clusters to more proinflammatory and leukocyte migration pathways, as determined by scRNA-Seq. Finally, glycolytic and hypoxia signatures were enriched in granulocytes infiltrating human PJI tissues compared with paired blood samples, suggesting that these pathways may contribute to the failure of immune-mediated biofilm clearance, since G-MDSC recruitment is also observed during human PJI (22, 23). Collectively, these studies suggest that cell-targeted metabolic reprogramming could prove beneficial for biofilm treatment in combination with antibiotics, with G-MDSC glycolysis representing an attractive candidate.

## Results

### scRNA-Seq identifies prominent glycolytic and hypoxia signatures in granulocytes throughout the course of PJI.

**Figure 1:**
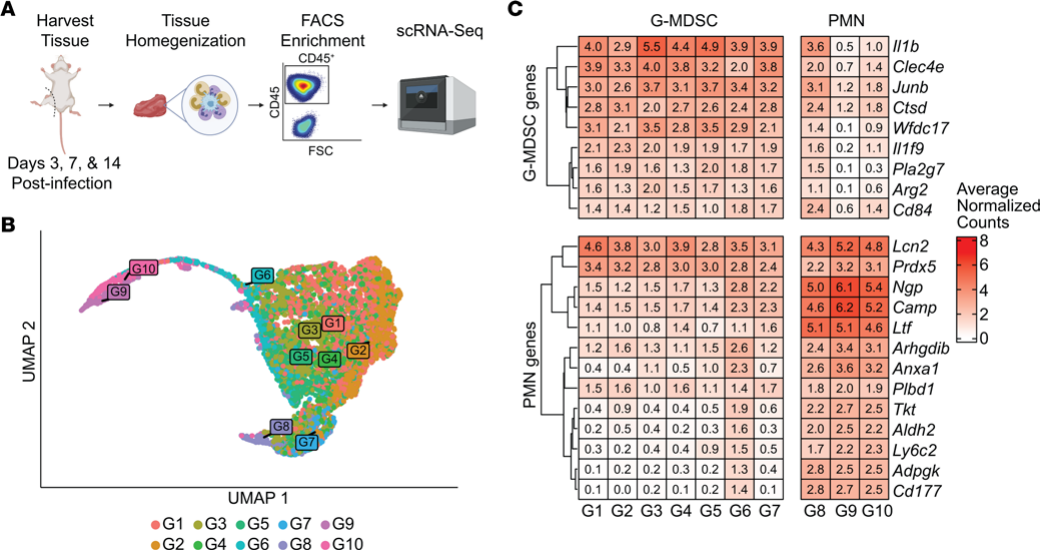
Longitudinal scRNA-Seq identifies G-MDSCs as the major cellular infiltrate at the site of PJI. (**A**) CD45^+^ cells were isolated at days 3, 7, and 14 after infection (*n* = 3,976, 3,975, and 2,111, respectively) using a mouse model of *S*. *aureus* PJI and submitted for scRNA-Seq (schematic created using BioRender). (**B**) UMAP of granulocytes used for scRNA-Seq analysis. G1 reflects the most abundant cluster with the fewest cells present in G10. (**C**) Granulocytes were identified as G-MDSCs or PMNs based on gene expression patterns.

Prior work has established that PJI in both humans and our mouse model is associated with G-MDSC recruitment, which contributes to the establishment of chronic infection (17, 22–24). G-MDSCs are a heterogenous population whose features tend to exhibit a degree of context dependence (21, 37). To characterize this heterogeneity and how it evolves over time, single-cell transcriptomics was performed using a mouse model of *S*. *aureus* PJI. CD45^+^ leukocytes were isolated from the joint tissue of C57BL/6J mice infected with *S*. *aureus* USA300 LAC at days 3, 7, and 14 after infection, and scRNA-Seq was performed (Figure 1A). Bioinformatic analyses revealed that most cells were granulocytic in character, with a total of 10 transcriptionally distinct clusters (Figure 1B) that were uniformly represented across the time points (Supplemental Figure 1; supplemental material available online with this article; https://doi.org/10.1172/JCI174051DS1). Furthermore, a distinction between granulocytes that appeared to be immature (G-MDSC–like) versus mature (PMN-like) was identified using published gene sets (Figure 1C) (37). Most infiltrating cells were G-MDSC–like, as reflected by the lower cluster numbers (G1–G7), whereas PMNs were less abundant (G8–G10), in agreement with prior flow cytometry data from our laboratory (17, 19, 21, 23, 24). We refer to these cells as G-MDSCs and PMNs throughout the remainder of the study.

**Figure 2:**
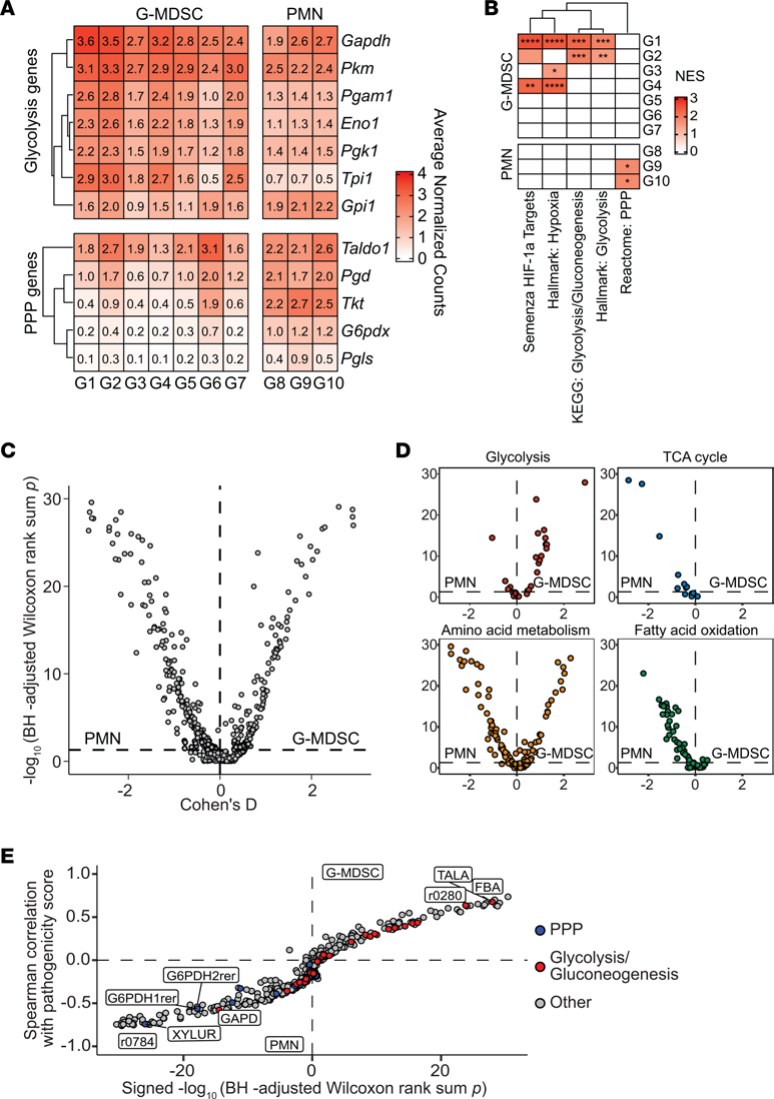
G-MDSCs infiltrating *S*. *aureus* PJI are characterized by strong glycolytic and hypoxia transcriptional signatures. (**A**) Expression of glycolytic and PPP genes in G-MDSC and PMN clusters identified by scRNA-Seq analysis. (**B**) Pathway analysis of differential expression data depicting glycolysis, hypoxia response, and PPP in G-MDSCs versus PMNs. *P < 0.05; **P < 0.01; ***P < 0.001; ****P < 0.0001, correlation-weighted Kolmogorov-Smirnov with Benjamini-Hochberg correction. (**C**) Metabolic modeling by COMPASS using scRNA-Seq expression data in G-MDSCs and PMNs with (**D**) specific pathways noted. (**E**) Identification of glycolytic and PPP genes and their relationship to predicted pathogenicity metabolic scores in G-MDSCs and PMNs.

**Figure 3:**
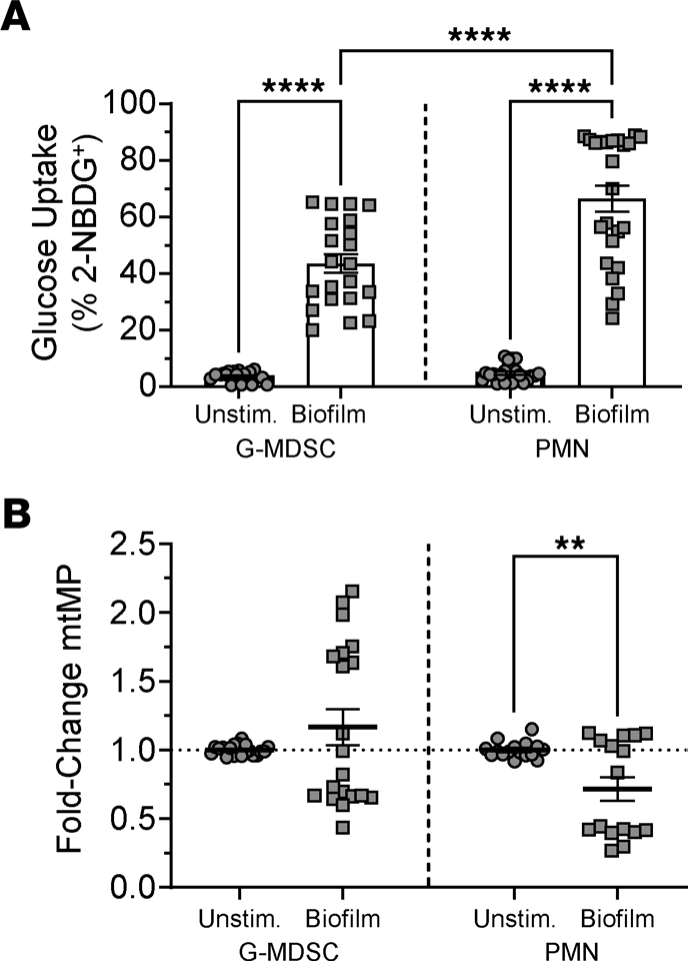
Glycolysis is increased in granulocytes following *S*. *aureus* biofilm exposure. Primary G-MDSCs and PMNs were either unstimulated or cocultured with *S*. *aureus* biofilm for 30 minutes, whereupon cells were stained with (**A**) 2-NBDG or (**B**) TMRM to monitor glucose uptake and mitochondrial transmembrane potential, respectively. (**A**) 2-NBDG data are represented as means ± SEM of 2-NBDG^+^ granulocytes. G-MDSC, *n* = 19 for unstimulated (unstim.); *n* = 21 for biofilm; PMN, *n* = 24/sample. ****P < 0.0001, 1-way ANOVA with Tukey’s correction. (**B**) TMRM data are represented as means ± SEM fold change in granulocytes cocultured with biofilm versus unstimulated cells. G-MDSC, *n* = 20/sample; PMN, *n* = 16/sample. **P < 0.01; unpaired, 2-tailed *t* test.

We next interrogated this data set to identify genes and pathways that were induced following PJI using differential expression testing followed by pathway analysis with gene set enrichment analysis (GSEA). At the gene expression level, many core glycolytic genes were significantly upregulated in G-MDSCs and PMNs (Figure 2A). Interestingly, genes involved in the PPP, which branches off glycolysis, were more enriched in PMNs compared with G-MDSCs (Figure 2A). At the pathway level, glycolysis and the hypoxia response through HIF1a were significantly increased in G-MDSCs, whereas the PPP was elevated in PMNs (Figure 2B). To complement these findings with an independent method, metabolic modeling with the COMPASS algorithm was performed (38). G-MDSCs and PMNs were independently analyzed with this approach prior to a statistical comparison of their resultant outputs (Supplemental Figure 2). Although predicted metabolic reactions were equally distributed between G-MDSCs and PMNs (Figure 2C), COMPASS analysis confirmed that glycolytic metabolism was significantly enriched in G-MDSCs, whereas fatty acid oxidation was more prominent in PMNs and amino acid metabolism was equivalent in both granulocyte populations (Figure 2D). Combining the predicted metabolic activities generated by COMPASS with the expression of G-MDSC and PMN gene sets allowed us to build a connection between gene expression and metabolic activity (Supplemental Figure 3B). Using this approach, the expression of G-MDSC and PMN genes, which we combined and quantified as the cellular pathogenicity and maturity scores (Supplemental Figure 3A), was strongly associated with many glycolytic and PPP reactions, respectively (Figure 2E). We found that lower pathogenicity scores (i.e., PMNs) were more linked with early reactions in the PPP (Figure 2E). To ensure that these findings could be recapitulated outside of an in silico environment, we utilized a flow cytometry–based strategy using 2-NBDG uptake as a proxy for glycolytic activity in granulocytes cocultured with *S*. *aureus* biofilm in vitro. 2-NBDG uptake was significantly increased in both G-MDSCs and PMNs after acute (30 minutes) exposure to biofilm (Figure 3A). In contrast, minimal changes in oxidative status were observed using the mitochondrial transmembrane potential dye TMRM (Figure 3B).

### Glycolysis is a key contributor to granulocyte ROS production in response to S. aureus biofilm.

**Figure 4:**
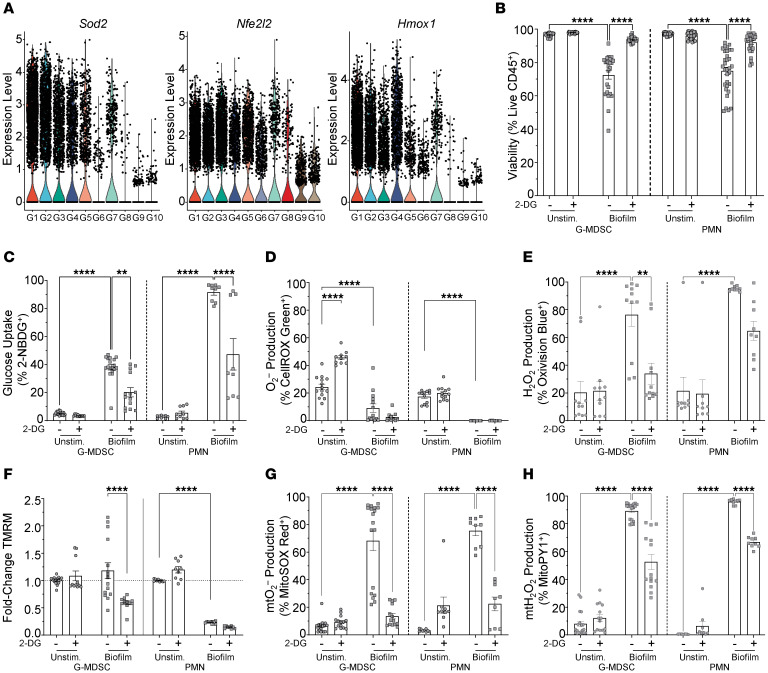
Granulocyte glycolysis induced by *S*. *aureus* biofilm is linked to ROS generation. (**A**) Expression levels of ROS-related genes in granulocyte clusters identified by scRNA-Seq in the mouse PJI model. (**B**–**H**) Primary G-MDSCs or PMNs were treated with either vehicle or 2-DG for 1 hour prior to *S*. *aureus* biofilm coculture for 30 minutes, whereupon granulocytes were evaluated for (**B**) cell viability (G-MDSC, *n* = 24/group; PMN, *n* = 32/group), (**C**) glucose uptake (2-NBDG) (G-MDSC, *n* = 18 for vehicle and *n* = 14 for 2-DG; PMN, *n* = 9/group), (**D**) O_2_^–^ (CellROX Green) (G-MDSC, *n* = 14 for vehicle and *n* = 10 for 2-DG; PMN, *n* = 12/group), (**E**) H_2_O_2_ (OxiVision) (G-MDSC, *n* = 11/group; PMN, *n* = 9/group), (**F**) mitochondrial transmembrane potential (mtMP; TMRM) (G-MDSC, *n* = 15 for vehicle and *n* = 11 for 2-DG; PMN, *n* = 9/group), (**G**) mtO_2_^–^ (MitoSOX) (G-MDSC, *n* = 18 for vehicle and *n* = 14 for 2-DG; PMN, *n* = 9/group), and (**H**) mtH_2_O_2_ (MitoPY) (G-MDSC, *n* = 18 for vehicle and *n* = 14 for 2-DG; PMN, *n* = 9/group). Results, excluding TMRM, are represented as means ± SEM of positively stained cells. TMRM data are represented as means ± SEM fold change in granulocytes cocultured with biofilm versus unstimulated cells. Viability data are represented as means ± SEM of viable cells. **P < 0.01; ****P < 0.0001, 1-way ANOVA with Tukey’s correction.

We next examined how glycolytic activity in granulocytes affected other metabolic features in response to *S*. *aureus* biofilm, namely ROS production. This was because scRNA-Seq identified several ROS-related genes that were significantly enriched in select granulocyte clusters in vivo, including *Sod2*, *Nfe2l2*, and *Hmox1* (Figure 4A). Granulocyte ROS is produced by the action of NADPH oxidases or mitochondrial metabolism through the electron transport chain. Glycolysis generates substrates that are required for both pathways; however, it was unclear how these may be interconnected in the context of *S*. *aureus* biofilm exposure. To address this potential crosstalk, granulocytes were treated with the glycolytic inhibitor 2-DG to assess how restricting glycolysis altered ROS responses to *S*. *aureus* biofilm. Not only did 2-DG attenuate glycolytic metabolism, as reflected by a significant decrease in the percentage of 2-NBDG^+^ cells (Figure 4C), but it also significantly reduced mitochondrial ROS (Figure 4G) as well as its conversion to hydrogen peroxide (H_2_O_2_) (Figure 4H) in both G-MDSCs and PMNs after biofilm coculture. Furthermore, mitochondrial membrane potential was also significantly reduced following 2-DG treatment in G-MDSCs but not PMNs in response to *S*. *aureus* biofilm (Figure 4F). The reason for reduced TMRM staining in PMNs following biofilm coculture is not known; however, one explanation may be cell-type selectivity, where a reduction in mitochondrial transmembrane potential forces PMNs to rely more heavily on glycolysis. This is supported by heightened 2-NBDG uptake in PMNs following biofilm coculture compared with G-MDSCs, where the increase was lower in magnitude (Figure 3A and Figure 4C). Superoxide production was significantly decreased in granulocytes after biofilm exposure compared with that in resting cells (Figure 4D), which was interpreted to reflect rapid conversion to H_2_O_2_ that was dramatically increased in this setting and was sensitive to 2-DG (Figure 4E). It is unclear why CellROX Green staining was increased in unstimulated G-MDSCs following 2-DG treatment, which was not observed in PMNs (Figure 4D). This may be unique to G-MDSCs, given their immune-suppressive characteristics, which are mediated by ROS (39, 40). This would suggest that constitutive glycolytic activity dampens ROS-generating pathways in G-MDSCs, which is supported by an earlier report documenting that the glycolytic metabolite phosphoenolpyruvate prevented excessive ROS production in G-MDSCs, but not PMNs (41). Interestingly, the metabolic changes resulting from 2-DG treatment coincided with increased granulocyte survival following biofilm exposure (Figure 4B). Collectively, these findings demonstrate the central role of glycolysis in metabolic programming of granulocyte ROS production in response to *S*. *aureus* biofilm.

### G-MDSCs utilize glycolysis for their immunosuppressive activity.

**Figure 5:**
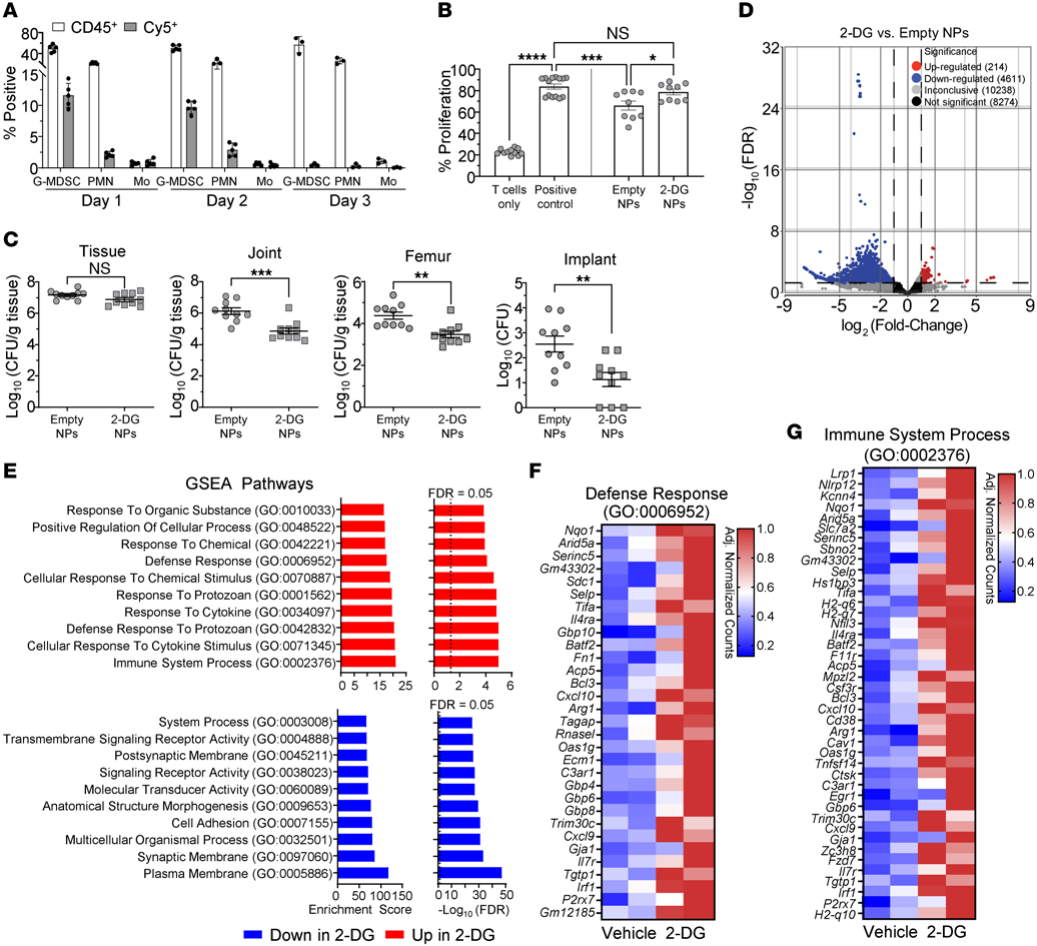
2-DG NP reduces G-MDSC–mediated immunosuppression, enhances proinflammatory responses, and improves biofilm clearance in vivo. (**A**) Mice received 1 injection of Cy5-labeled NP in the soft tissue surrounding the infected joint at day 3 after infection, whereupon NP uptake was quantified on 3 consecutive days by flow cytometry. Results are represented as the percentage of Cy5^+^ cells for each leukocyte infiltrate. Data are represented as means ± SD. Days 1 and 2, *n* = 5/group; day 3, *n* = 3/group. (**B** and **C**) Mice received a single dose of empty or 2-DG NP at day 3 after infection, whereupon G-MDSCs were isolated from the soft tissue surrounding the infected joint at day 14 by FACS to assess (**B**) antiinflammatory activity by a T cell–suppression assay (data are represented as means ± SEM; T cells only, *n* = 11; positive control, *n* = 14; empty NPs and 2-DG NPs, *n* = 9; *P < 0.05; ***P < 0.001; ****P < 0.0001, 1-way ANOVA with Tukey’s correction) and (**C**) bacterial burden in the tissue, joint, femur and implant (data are represented as means ± SEM; *n* = 10 /group; **P < 0.01; ***P < 0.001; unpaired 2-tailed *t* test). (**D**–**G**) CD45^+^Ly6G^+^ granulocytes were recovered by FACS from the soft tissue of mice receiving 2-DG or empty NPs at day 7 after infection (4 days following NP injection; *n* = 3 mice/group for each of 2 biological replicates). (**D**) Volcano plot of differentially expressed genes, (**E**) enriched pathways identified by GSEA, and heatmaps of genes involved in (**F**) defense response and (**G**) immune system process pathways.

To examine the importance of G-MDSC glycolysis in shaping *S*. *aureus* biofilm development in vivo, a NP was used to deliver 2-DG at the site of PJI. Local delivery was favored over systemic administration due to the confined nature of PJI in both humans and our mouse model (22, 24) in addition to potential off-target effects of 2-DG with systemic dosing. Our NP formulation lacked a targeting moiety, as no unique surface marker has been identified for G-MDSCs because of their similarity to PMNs. However, since G-MDSC infiltrates dramatically outnumber PMNs during PJI (17, 19, 23, 24), we predicted that NP internalization would be skewed toward G-MDSCs. Indeed, this was observed, where G-MDSCs exhibited the greatest uptake of Cy5-labeled NPs in vivo compared with PMNs and monocytes (Figure 5A). Cy5 signals declined over time, in agreement with our prior report with oligomycin-loaded NPs, although biological effects were sustained (16). To assess the impact of G-MDSC glycolysis on PJI outcome, mice received 1 injection of either empty or 2-DG NPs directly into the soft tissue surrounding the joint on day 3 after infection. Importantly, this posttreatment paradigm allowed for early bacterial expansion, better modeling a translational scenario. Animals were euthanized 11 days following NP administration (day 14 after infection) to assess bacterial burden and G-MDSC functional activity using a T cell–suppression assay. As we previously reported, G-MDSCs recovered from *S*. *aureus* PJI significantly inhibited T cell proliferation (empty NPs); however, treatment with 2-DG NPs ablated this inhibitory activity (Figure 5B). Furthermore, 2-DG NP administration significantly reduced bacterial burden in the joint, femur, and implant (Figure 5C). Importantly, 2-DG did not affect *S*. *aureus* planktonic (Supplemental Figure 4A) or biofilm (Supplemental Figure 4D) growth at the same concentration delivered by NPs in vivo. Furthermore, 2-DG had no impact on *S*. *aureus* metabolism, as glycolysis, TCA cycle, PPP, and nucleotide metabolites were similar between 2-DG and vehicle-treated bacteria under both growth conditions (Supplemental Figure 4, B, C, E, and F, and Supplemental Table 2). Collectively, these findings demonstrate that the reduction in bacterial burden with 2-DG treatment resulted from immune modulation and likely not direct effects on *S*. *aureus*.

To further assess how 2-DG NP treatment altered granulocyte activation during PJI, bulk RNA-Seq was performed on Ly6G^+^ granulocytes recovered from mice receiving 2-DG versus empty NPs. Bulk RNA-Seq was utilized instead of scRNA-Seq, since only a subpopulation of G-MDSCs internalized 2-DG NPs (Figure 5A) and this heterogeneity could not be resolved at the single-cell level. Furthermore, G-MDSCs recovered from 2-DG NP–treated mice could no longer suppress T cell activation (Figure 5B) despite only a fraction of cells being targeted, and the abundance of G-MDSCs at the site of PJI compared with the lower number of PMNs suggested that bulk sequencing would accurately report changes in cellular activation status. Differential expression analysis revealed 214 upregulated and 4,611 downregulated genes in granulocytes recovered from 2-DG NP–treated mice compared with empty NPs (Figure 5D). Pathway analysis identified significant increases in genes involved in defense response, cytokine response, and response to protozoa in granulocytes recovered from mice receiving 2-DG NPs, providing further support that glycolysis promotes G-MDSC antiinflammatory properties. Heatmaps of defense response and immune system process pathways revealed increased expression of inflammasome (*Nlrp12* and *P2rx7*) and type I interferon–induced (*Gbps*, *Oas1g*, *Irf1* and *Rnasel*) and inflammatory (*Ccxl9*, *Cxcl10*, and *Tnfsf14*) genes in granulocytes recovered from 2-DG NP–treated animals (Figure 5, F and G). In contrast, downregulated pathways were more generalized in scope, representing cell membrane and adhesion along with signal transduction (Figure 5E). Given that G-MDSCs internalized most 2-DG NPs, which inhibited their suppressive activity and promoted proinflammatory transcriptional profiles, these findings suggest that glycolytic metabolism plays an important role in the ability of G-MDSCs to promote *S*. *aureus* biofilm persistence in vivo.

### Granulocyte HIF1a is critical for promoting S. aureus PJI.

**Figure 6:**
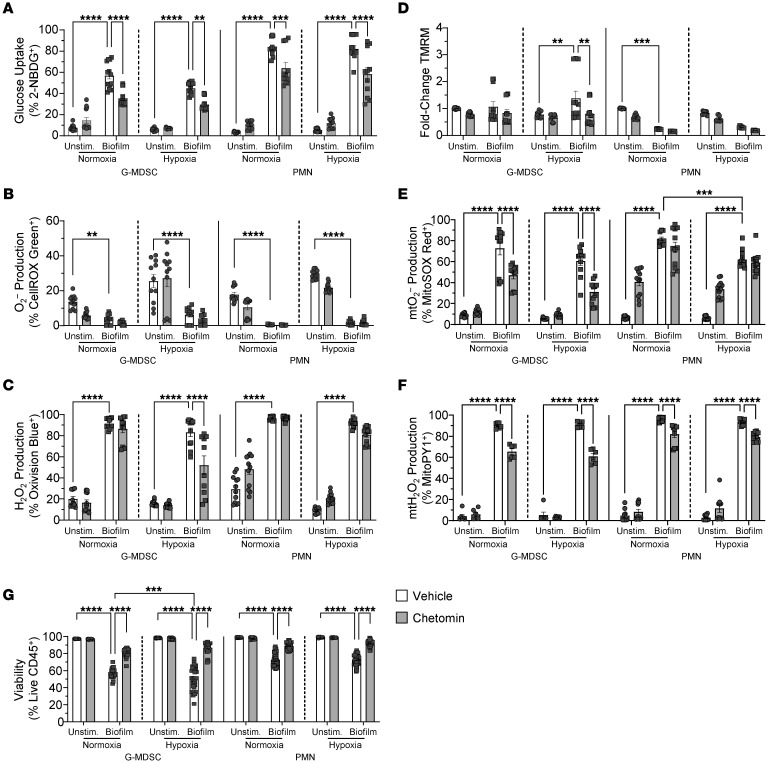
Inhibition of HIF1a signaling attenuates granulocyte ROS production in response to *S*. *aureus* biofilm. Primary G-MDSCs or PMNs were treated with either chetomin or vehicle for 1 hour under normoxia or hypoxia (1% O_2_) prior to coculture with *S*. *aureus* biofilm for 30 minutes under the same oxygen conditions, whereupon cells were stained with (**A**) 2-NBDG (glucose uptake) (G-MDSC, *n* = 13/group; PMN, *n* = 11/group), (**B**) CellROX Green (O_2_^–^) (G-MDSC, *n* = 11/group; PMN, *n* = 12/group), (**C**) OxiVision (H_2_O_2_) (G-MDSC, *n* = 11/group, except for hypoxia/chetomin/biofilm, *n* = 10; PMN, *n* = 12/group), (**D**) TMRM (mitochondrial transmembrane potential; mtMP) (G-MDSC, *n* = 12/group; PMN, *n* = 9/group), (**E**) MitoSOX (mtO_2_^–^) (G-MDSC, *n* = 11/group; PMN, *n* = 12/group), and (**F**) MitoPY (mtH_2_O_2_) (G-MDSC, *n* = 6/group; PMN, *n* = 10/group). (**G**) Cell viability data are represented as the percentage of live CD45^+^ cells (G-MDSC, *n* = 28/group, except for hypoxia/chetomin/biofilm, *n* = 27; PMN, *n* = 40/group). Results are represented as means ± SEM of positively stained or fold change (TMRM) in granulocytes cocultured with biofilm versus unstimulated cells under normoxia or hypoxia. **P < 0.01; ***P < 0.001; ****P < 0.0001, 2-way ANOVA with Tukey’s correction.

In addition to glycolysis, the hypoxia response and HIF1a transcription factor pathway were among the most significantly enriched pathways in G-MDSC subsets (Figure 2B). Bone is an intrinsically low-oxygen environment that is exacerbated by infection, where O_2_ levels drop to approximately 1% (42). HIF1a is a master regulator of the cellular response to hypoxia and plays a key role in immune regulation (43). Although prior work has shown that HIF1a promotes leukocyte proinflammatory activity and bacterial clearance in models of planktonic infection (44–46), we explored whether it may be detrimental in the context of biofilm infection by augmenting G-MDSC glycolytic activity. To first address this possibility in vitro, granulocyte-biofilm cocultures were treated with chetomin, an inhibitor of HIF-mediated signaling (47). In addition, parallel assessments were made under hypoxia (1% O_2_) to model oxygen levels in infected bone (42). Like 2-DG, the increases in 2-NBDG uptake and mitochondrial H_2_O_2_ elicited by *S*. *aureus* biofilm in G-MDSCs and PMNs were partly dependent on functional HIF signaling (Figure 6, A and F). Interestingly, total H_2_O_2_ (Figure 6C), mitochondrial membrane potential (Figure 6D), and mitochondrial ROS production (Figure 6E) were significantly decreased by chetomin in G-MDSCs under hypoxia, whereas these measures were unaffected by chetomin in PMNs. Again, significant decreases in superoxide were detected in both granulocyte populations after biofilm exposure (Figure 6B), which was attributed to rapid conversion to H_2_O_2_ (Figure 6C). Chetomin also increased granulocyte viability following *S*. *aureus* biofilm coculture compared with vehicle-treated cells (Figure 6G) without affecting *S*. *aureus* survival under planktonic or biofilm growth conditions at the same concentration (Supplemental Figure 4, G–I). In general, similar metabolic changes were observed when G-MDSC–biofilm cocultures were incubated under normoxic or hypoxic conditions, except for mitochondrial membrane potential and total H_2_O_2_ production, whereas PMN responses were less altered, with 2-NBDG uptake being the most affected (Figure 6). This is likely influenced by the microaerobic environment created by the biofilm even in oxygen-replete conditions. Importantly, hypoxia did not dramatically alter *S*. *aureus* biofilm growth in vitro (Supplemental Figure 4J).

**Figure 7:**
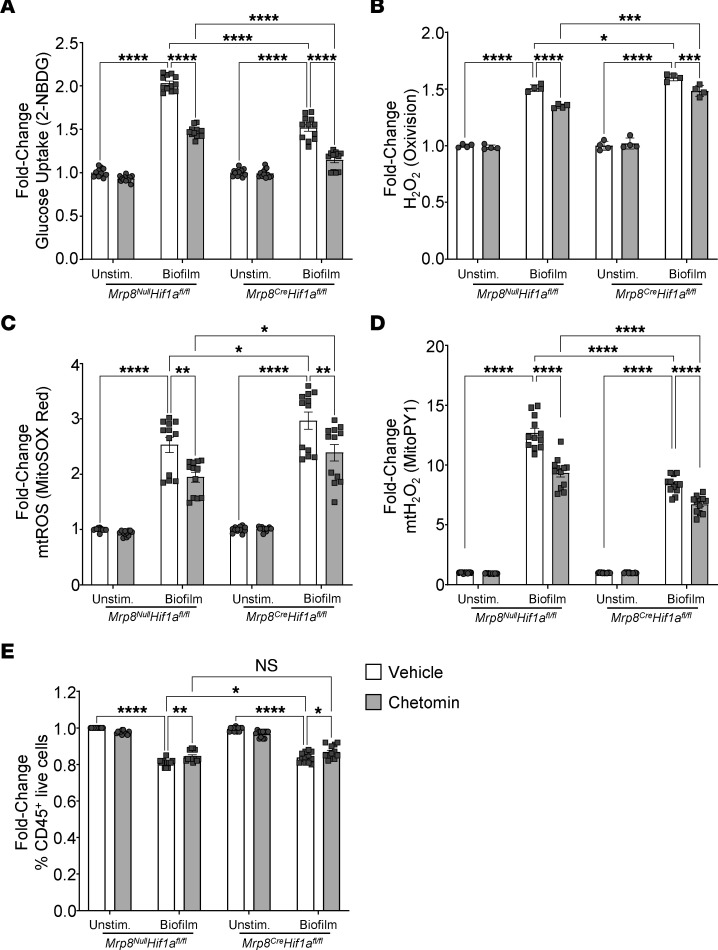
G-MDSC metabolic responses to *S*. *aureus* biofilm are partially HIF1a dependent. Primary G-MDSCs from *Mrp8^Null^Hif1a^fl/fl^* and *Mrp8^Cre^Hif1a^fl/fl^* mice were treated with either chetomin or vehicle for 1 hour prior to coculture with *S*. *aureus* biofilm for 30 minutes, whereupon cells were stained with (**A**) 2-NBDG (glucose uptake) (*n* = 12/group), (**B**) OxiVision (H_2_O_2_) (*n* = 4/group), (**C**) MitoSOX (mtO_2_^–^) (*n* = 12/group), (**D**) MitoPY (mtH_2_O_2_) (*n* = 12/group), and (**E**) a UV live/dead fixable dye (viability) (*n* = 12/group). Results are expressed as the fold-change of each genotype normalized to resting cells. Results, excluding Oxivision staining, are represented as means ± SEM. Oxivision data are represented as means ± SD.*P < 0.05; **P < 0.01; ***P < 0.001; ****P < 0.0001, 2-way ANOVA with Tukey’s correction.

**Figure 8:**
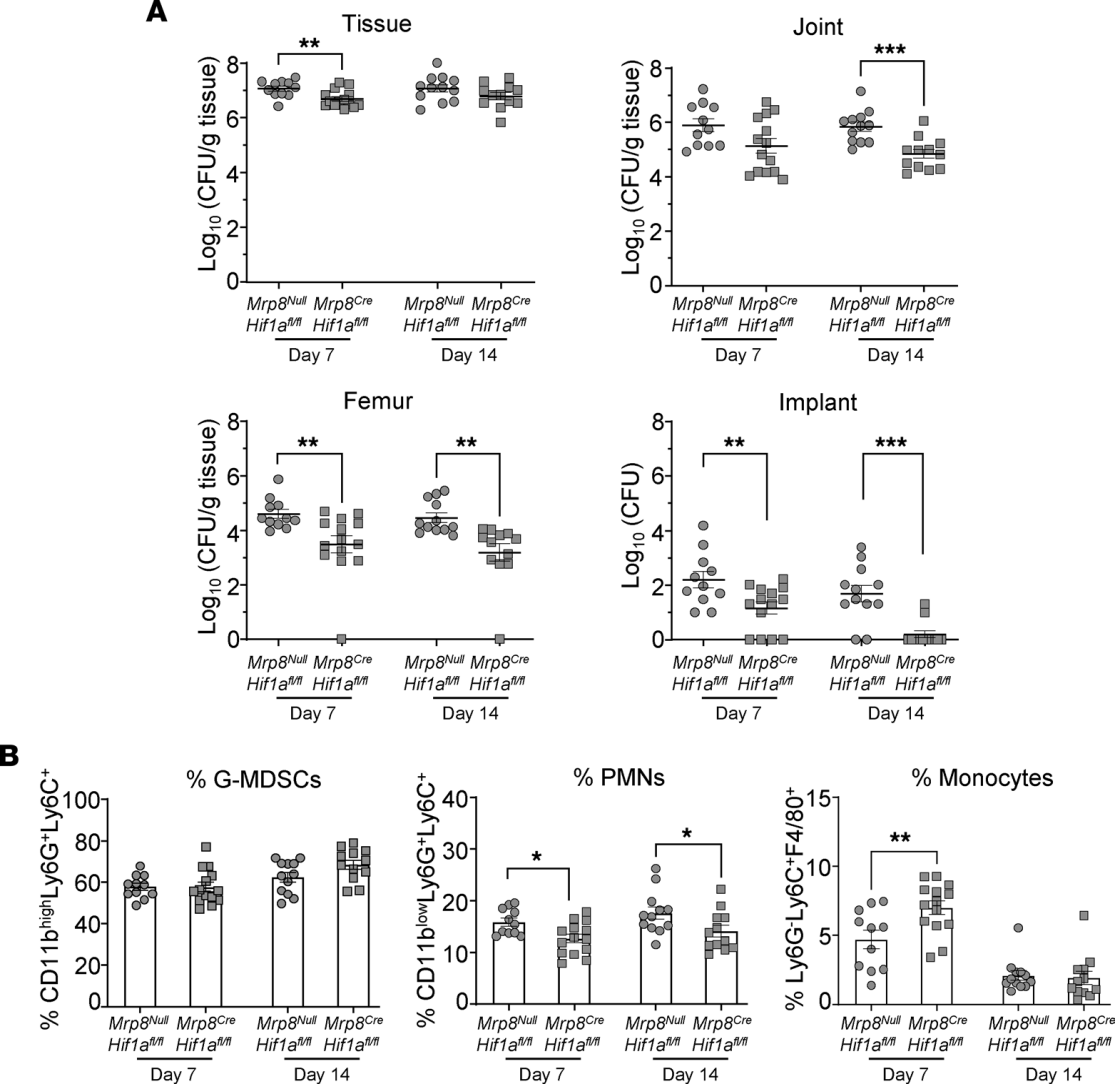
*Mrp8Hif1a* conditional KO mice exhibit improved infection outcomes. *Mrp8^Null^Hif1a^fl/fl^* and *Mrp8^Cre^Hif1a^fl/fl^* mice were infected with *S*. *aureus*, whereupon (**A**) bacterial burden in the tissue, joint, femur, and on the implant and (**B**) immune infiltrates in the soft tissue surrounding the infected joint were assessed at days 7 (*n* = 11 for *Mrp8^Null^Hif1a^fl/fl^*; *n* = 14 for *Mrp8^Cre^Hif1a^fl/fl^*) and 14 (*n* = 12/group) after infection. Data are represented as mean ± SEM. *P < 0.05; **P < 0.01; ***P < 0.001, unpaired 2-tailed *t* test.

To determine the role of HIF1a in granulocytes during PJI, *Mrp8^Cre^Hif1a^fl/fl^* conditional KO mice were generated, since global *Hif1a* deletion is embryonic lethal (48). The *Mrp8*-Cre driver targets gene deletion to granulocytes (49), which includes G-MDSCs but also PMNs based on their relatedness. Characterization of G-MDSCs from *Mrp8^Cre^Hif1a^fl/fl^* mice revealed reduced *Hif1a* mRNA and protein expression (Supplemental Figure 5, A and B) and no induction of Hif1a-dependent genes under hypoxia (Supplemental Figure 5C). Targeting specificity was demonstrated by similar *HIF1a* mRNA, protein, and Hif1a-dependent gene expression in macrophages from *Mrp8^Cre^Hif1a^fl/fl^* and *Mrp8^Null^Hif1a^fl/fl^* animals (Supplemental Figure 5, A–C). After biofilm coculture in vitro, *Mrp8^Cre^Hif1a^fl/fl^* G-MDSCs displayed significant reductions in glucose uptake compared with WT cells (*Mrp8^Null^Hif1a^fl/fl^*) (Figure 7A). However, 2-NBDG uptake in *Mrp8^Cre^Hif1a^fl/fl^* G-MDSCs was further reduced by chetomin (Figure 7A), reflecting either effects on residual HIF1a expression (Supplemental Figure 5B) and/or the involvement of HIF1a-independent mechanisms. No dramatic differences in total H_2_O_2_ (Figure 7B) or cell viability (Figure 7E) were observed between *Mrp8^Cre^Hif1a^fl/fl^* and *Mrp8^Null^Hif1a^fl/fl^* G-MDSCs, whereas mitochondrial ROS (mtROS) (Figure 7C) and mtH_2_O_2_ (Figure 7D) displayed divergent responses in *Mrp8^Cre^Hif1a^fl/fl^* G-MDSCs. In vivo, bacterial burden was significantly decreased in *Mrp8^Cre^Hif1a^fl/fl^* mice at days 7 and 14 after infection in various PJI tissues compared with *Mrp8^Null^Hif1a^fl/fl^* littermates, but was most pronounced in the femur and implant (Figure 8A). This coincided with a significant reduction in PMN influx and increased monocyte recruitment at the site of infection (Figure 8B) that was generally reflected by absolute cell counts (Supplemental Figure 6).

**Figure 9:**
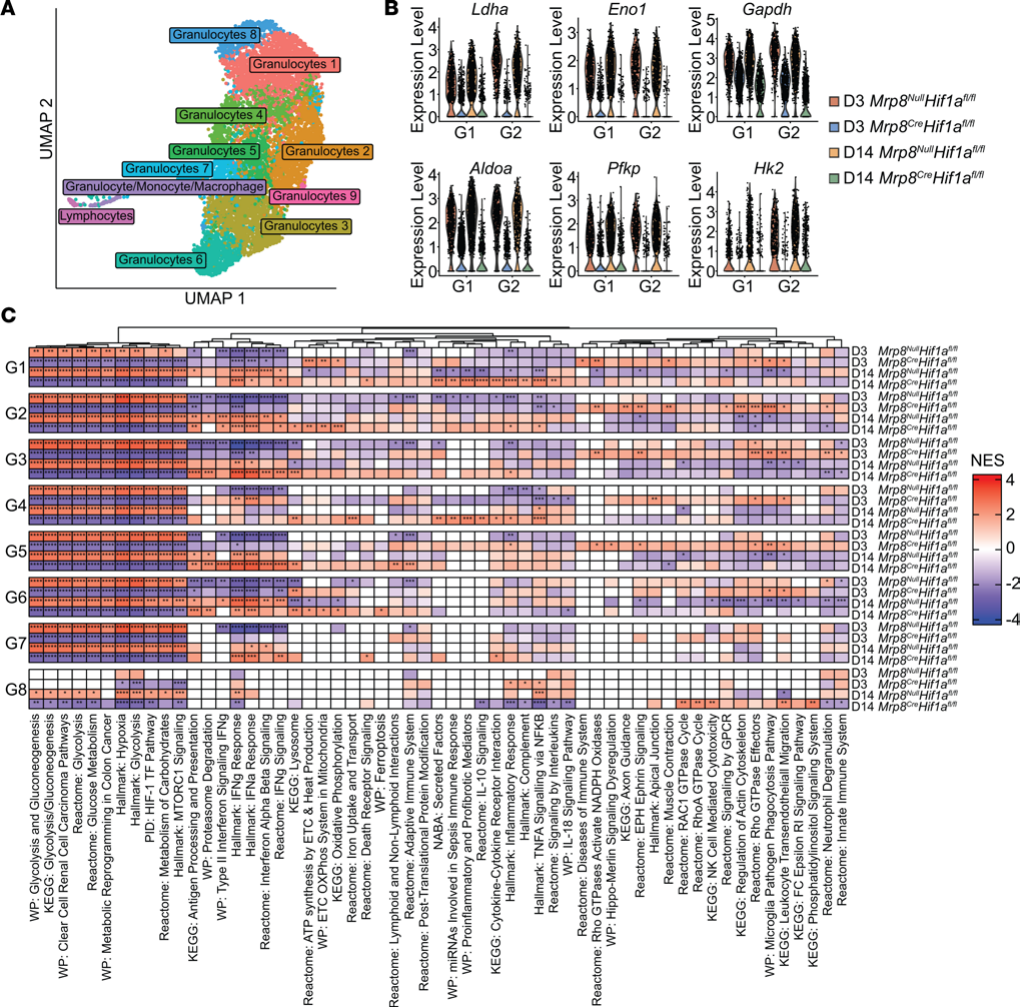
Conditional *Hif1a* deletion in granulocytes significantly attenuates the expression of glycolytic genes concomitant with increased proinflammatory profiles. (**A**) CD45^+^ cells were recovered by FACS from *Mrp8^Null^Hif1a^fl/fl^* and *Mrp8^Cre^Hif1a^fl/fl^* mice (*n* = 4 mice/group) at days 3 (*n* = 1,629 and 2,366 cells, respectively) and 14 (*n* = 2,725 and 1,991 cells, respectively) after *S*. *aureus* PJI for single-cell sequencing. The resultant data sets were integrated and clustered using a UMAP procedure. (**B**) Expression levels of core glycolytic genes in the most abundant granulocyte clusters (G1 and G2). (**C**) Pathway analysis using differentially expressed genes identified in *Mrp8^Null^Hif1a^fl/fl^* and *Mrp8^Cre^Hif1a^fl/fl^* granulocyte clusters. *P < 0.05; **P < 0.01; ***P < 0.001; ****P < 0.0001, correlation-weighted Kolmogorov-Smirnov with Benjamini-Hochberg correction.

To assess how *Hif1a* loss in granulocytes affected gene expression in vivo, CD45^+^ cells were isolated from *Mrp8^Cre^Hif1a^fl/fl^* and *Mrp8^Null^Hif1a^fl/fl^* mice at days 3 and 14 after infection for scRNA-Seq. These time points were selected to evaluate how HIF1a affects leukocyte responses during planktonic infection (day 3) versus mature biofilm formation (day 14) (16). UMAP analysis confirmed our earlier scRNA-Seq data where granulocyte clusters were dominant (Figure 9A) and, importantly, the numbers of cells within each granulocyte cluster from *Mrp8^Cre^Hif1a^fl/fl^* and *Mrp8^Null^Hif1a^fl/fl^* mice were equally represented across both time points (Supplemental Figure 7). Glycolysis was significantly decreased in all *Mrp8^Cre^Hif1a^fl/fl^* granulocyte clusters at both time points, as demonstrated by differential expression of genes encoding glycolytic enzymes (Figure 9B), pathway analysis (Figure 9C), and COMPASS (Supplemental Figures 8 and 9). Pathway analysis suggested that *Mrp8^Cre^Hif1a^fl/fl^* granulocytes displayed a more proinflammatory phenotype, as revealed by significantly increased IFN signaling, TNF signaling via NF-κB, and complement pathways (Figure 9C). These changes were most evident at day 14, whereas *Mrp8^Cre^Hif1a^fl/fl^* granulocytes at day 3 after infection exhibited increased expression of genes involved in cytoskeleton and leukocyte migration along with Rho GTPase activation of NADPH oxidases (Figure 9C). Collectively, these findings demonstrate that during *S*. *aureus* PJI, HIF1a contributes to enhanced glycolytic metabolism in granulocytes that paradoxically diminishes the expression of proinflammatory genes. This appears to be unique to granulocytes in the context of biofilm infection compared with the well-described effects of HIF1a on promoting macrophage proinflammatory responses (43).

### Granulocytes from PJI patients display glycolytic and hypoxia signatures.

**Figure 10:**
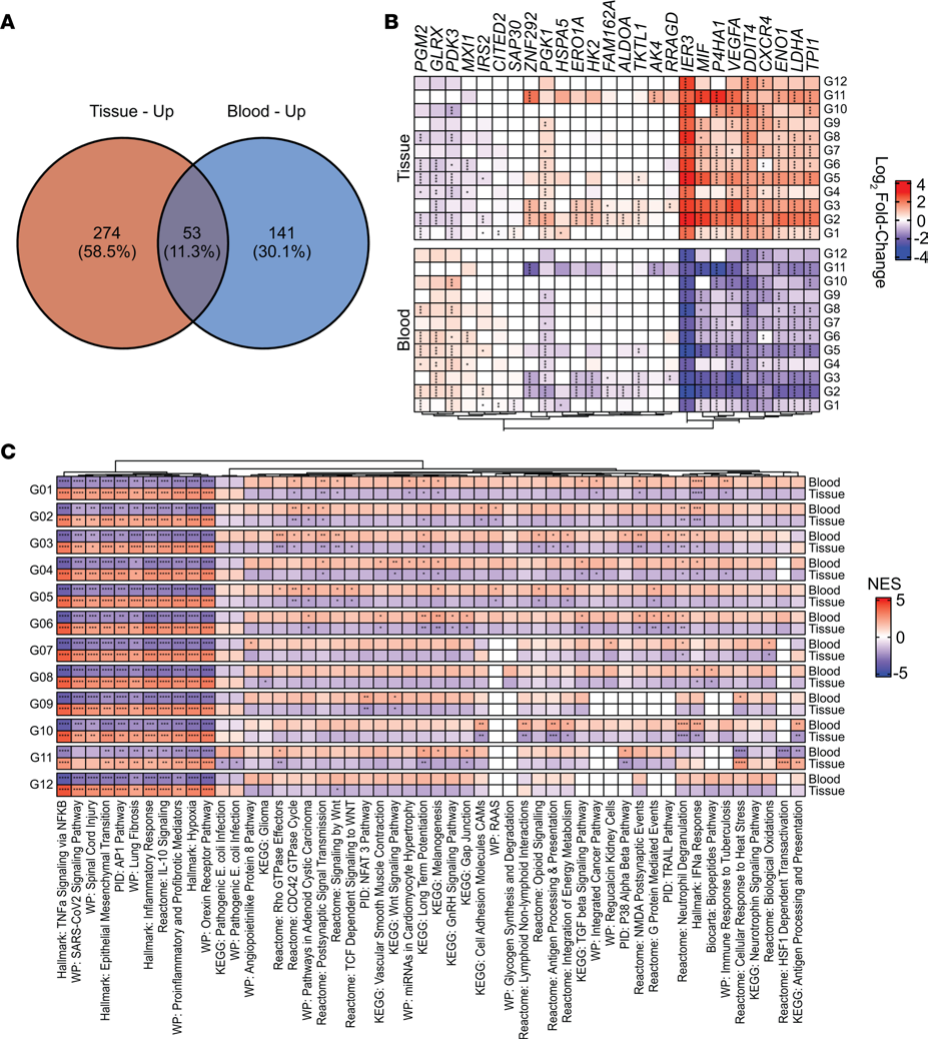
Leukocytes from human PJI patients exhibit significant transcriptional changes in glycolysis and the hypoxia response. scRNA-Seq was performed on cells isolated from the blood and tissues of patients with PJI. *n* = 3 patients. Subject 1: *n* = 4,825 (blood) and *n* = 4,575 (tissue); subject 2: *n* = 4,940 (blood) and *n* = 6,936 (tissue); subject 5: *n* = 1,573 (blood) and *n* = 2,215 (tissue). Differential expression testing was used to identify (**A**) characteristic pathway utilization that was either shared or unique to each sample origin, (**B**) changes in glycolytic and hypoxia response genes (*P < 0.05; **P < 0.01; ***P < 0.001; ****P < 0.0001; MAST with Bonferroni’s correction), and (**C**) top differentially regulated pathways (*P < 0.05; **P < 0.01; ***P < 0.001; ****P < 0.0001, correlation-weighted Kolmogorov-Smirnov with Benjamini-Hochberg correction) between granulocytes from the blood versus infected tissue.

To determine whether the glycolytic and HIF1a bias observed in our mouse model of PJI extended to human disease, scRNA-Seq was performed on tissues collected from patients with PJI along with paired blood samples. Blood leukocytes were used as a comparator for each subject given the fact that PJI is localized with minimal peripheral involvement (22). The demographics of the 3 subjects included in this study are presented in Supplemental Table 1. Integration of patient scRNA-Seq data revealed a predominance of granulocyte clusters (Supplemental Figure 10), in agreement with our mouse model and confirming prior studies where G-MDSC and PMN infiltrates were prevalent in human PJI tissues (22, 23). Bioinformatic analyses supported that the response to PJI was highly localized, as tissue samples displayed more gene and pathway activity relative to the blood (Figure 10A). Importantly, glycolysis and the hypoxia response were significantly increased in granulocyte clusters from PJI subjects, as evident from gene signatures (Figure 10B) and pathway data (Figure 10C) from the integrated data set, validating the findings in our mouse PJI model (Figure 2). IL-10 signaling was elevated in granulocytes from human PJI tissues, which is a major product of G-MDSCs that inhibits leukocyte proinflammatory activity to promote PJI persistence (17, 19). Interestingly, several pathways were significantly increased in peripheral blood granulocytes compared with cells that had invaded the infection site, including WNT signaling, Rho and Cdc42 GTPases, G protein–mediated events, and IFN-α response (Figure 10C).

**Figure 11:**
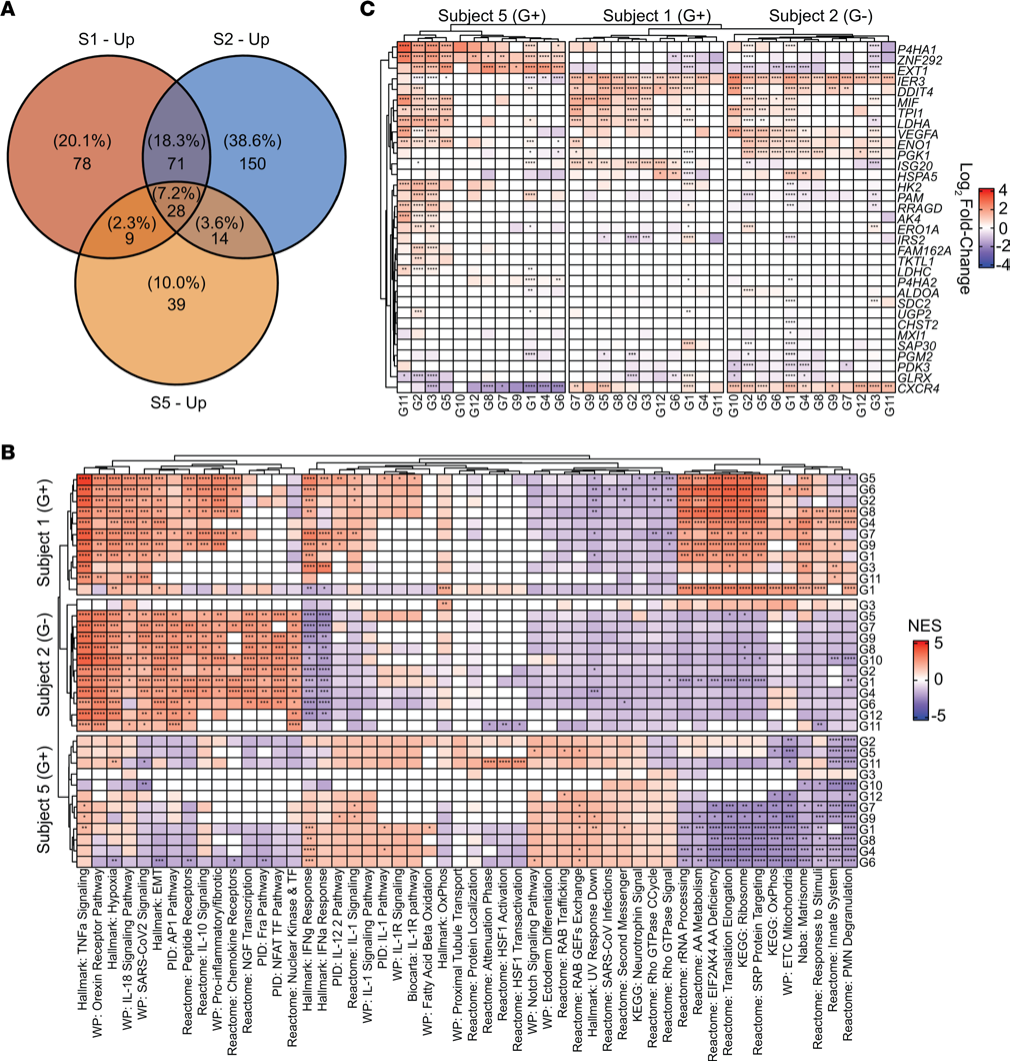
Granulocyte transcriptional profiles during human PJI are not linked to the inciting pathogen. scRNA-Seq data from human PJI patients were stratified by subject to examine potential pathogen-specific changes. Gram-positive, subjects 1 and 5; Gram-negative, subject 2. (**A**) Proportion of pathway overlap between patient samples. (**B**) Top pathways in each subject based on enrichment statistics. *P < 0.05; **P < 0.01; ***P < 0.001; ****P < 0.0001, correlation-weighted Kolmogorov-Smirnov with Benjamini-Hochberg correction. (**C**) Expression of genes involved in glycolysis and the hypoxia response. *P < 0.05; **P < 0.01; ***P < 0.001; ****P < 0.0001, MAST with Bonferroni’s correction.

Since our PJI subjects represented 2 Gram-positive (*S*. *aureus*) and 1 Gram-negative (*Stenotrophomonas maltophilia*) species, we next determined whether unique transcriptional signatures were present for each pathogen class. In general, only a limited number of pathways were shared between the 3 subjects, with each exhibiting largely distinct signatures (Figure 11A). The only transcriptional profiles that were largely conserved for the 2 *S*. *aureus* samples compared with the Gram-negative infection were increased IFN-α/γ response and several IL-1 signaling pathways (Figure 11B). Otherwise, unique pathway expression patterns were observed for each patient sample (Figure 11B). Interestingly, the expression of several glycolytic and hypoxia response genes was significantly increased across the granulocyte clusters of all 3 subjects (Figure 11C), supporting that glycolysis and HIF1a are core transcriptional platforms during PJI regardless of the inciting pathogen. Collectively, these findings demonstrate the acquisition of glycolytic and hypoxia signatures in granulocytes once they have exited the bloodstream into the site of PJI, highlighting the highly localized nature of these infections. The conserved glycolytic and hypoxia profiles in patient samples, combined with our mechanistic studies in the mouse PJI model, suggest these pathways likely play a critical role in biofilm persistence.

## Discussion

This report demonstrates that glycolytic metabolism and the HIF1a transcription factor pathway contribute to the pathologic immune suppression that is characteristic of granulocytes within the PJI milieu. This was supported by several lines of evidence including (a) increased expression of several glycolytic and hypoxia response genes in G-MDSCs in vivo that was reinforced by pathway-level analyses using several independent computational methods; (b) ability of 2-DG, delivered at the site of PJI using an NP paradigm, to significantly attenuate the inhibitory action of G-MDSCs on T cell proliferation and augment proinflammatory mediator expression; (c) critical role of granulocyte HIF1a in promoting *S*. *aureus* persistence by inhibiting the expression of several proinflammatory pathways; and (d) conservation of glycolytic and hypoxia signatures in granulocytes infiltrating human PJI tissues with the mouse model, reflecting a core metabolic platform elicited by biofilm infection. Our data showing that glycolysis is critical for G-MDSC antiinflammatory activity is an important advance, since both glycolysis and HIF1a promote proinflammatory activity in PMNs and macrophages (43). This highlights the distinct effector functions elicited by glycolysis in diverse microenvironments and potential unique responses elicited by biofilm growth.

Because of the strong glycolytic profile of granulocytes in response to *S*. *aureus* biofilm, our goal was to attenuate this metabolic program in G-MDSCs to diminish their antiinflammatory properties without significantly affecting glycolysis in PMNs and monocytes/macrophages that is important for their antimicrobial activity. To achieve this, we leveraged a local delivery approach with 2-DG–loaded NPs. Although it was not possible to selectively target NPs to G-MDSCs because of the lack of a unique marker with PMNs, the fact that G-MDSCs are the most abundant leukocyte infiltrate during PJI (~70%) suggested that NP uptake by other leukocytes might be limited. Indeed, this was observed, where the majority of 2-DG NPs were internalized by G-MDSCs, which reduced their suppressive activity and increased proinflammatory gene expression concomitant with lower bacterial burden in vivo. The relationship between decreased G-MDSC activity and lower bacterial abundance has been a recurrent theme in the PJI model, revealing the critical role of G-MDSCs in dictating infectious outcome (16, 17, 19, 23, 24). Of note, the effect of glycolysis in PMNs during PJI is likely complicated, since our prior work has revealed time-dependent alterations in their inflammatory properties. Specifically, PMNs had no effect on T cell proliferation during acute infection (i.e., day 3); however, beyond this interval (days 7–28) PMNs acquired potent T cell–suppressive activity (21). Therefore, although PMNs have classically been considered to exert antimicrobial activity through a glycolytic program, this may be altered in the context of chronic biofilm infection. Despite these caveats, glycolysis in PMNs was likely minimally affected by 2-DG, since they displayed limited NP uptake. Importantly, the amount of 2-DG delivered would not completely inhibit glycolysis, especially given the fact that 2-DG NPs were only administered once after infection was established (i.e., day 3) and effects on G-MDSC activity and *S*. *aureus* burden were not examined until 11 days later (i.e., day 14 after infection). This extended interval suggests that effects may be occurring at the epigenetic level based on the known longevity of epigenetic changes on immune cell function (50–53). This is also supported by our finding that G-MDSCs recovered from mice receiving 2-DG NPs retained their phenotype in vitro over the 3-day T cell–suppression assay. Indeed, many reports have established the connection between metabolic byproducts and epigenetic marks (51, 53, 54). Glycolysis is a major source of pyruvate, which can be converted to lactate or acetate, both of which have been shown to alter gene expression in immune cells by acting as substrates for epigenetic remodeling (19, 53, 54). Furthermore, transcriptomic analysis reinforced that inhibiting glycolysis in granulocytes with 2-DG NPs transformed cells into a more inflammatory phenotype typified by increased expression of genes involved in cytokine and immune-defense pathways. Interestingly, 2-DG had no effect on the expression of genes encoding glycolytic enzymes, suggesting that glycolytic byproducts are likely important for promoting the antiinflammatory properties of G-MDSCs. Although glycolysis and lactate production are important for *S*. *aureus* persistence during infection (19, 55, 56), our findings revealed that 2-DG treatment had no effect on *S*. *aureus* growth or metabolism under either planktonic or biofilm conditions, suggesting that 2-DG mainly influences host metabolism to attenuate bacterial burden during PJI. A recent study reported that glycolysis was important for driving G-MDSC maturation into PMNs in a mouse model of systemic *S*. *aureus* infection, although this had no effect on bacterial burden (57). However, these observations were made in MDSCs distant from the infection site and are at odds with our data showing that glycolysis in G-MDSCs promotes their antiinflammatory activity.

Our initial scRNA-Seq studies revealed an enrichment of genes involved in the hypoxia response alongside glycolysis, including *Hif1a*. It is well established that HIF1a can augment glycolytic metabolism for adapting to oxygen-limiting environments (43). Furthermore, prior work has established that *S*. *aureus*–infected bone is hypoxic (42), which would promote HIF1a stabilization. Based on the interplay between glycolysis and HIF1a, coupled with the observation that glycolysis was critical for promoting G-MDSC antiinflammatory activity, we next determined whether HIF1a influenced the phenotype of G-MDSCs during PJI. This was accomplished by engineering *Hif1a* conditional KO mice where the gene was targeted in granulocytes using *Mrp8^Cre^*. As with our NP approach, this strategy targeted HIF1a in both G-MDSCs and PMNs. However, as mentioned earlier, most granulocytes at the site of PJI are G-MDSCs and PMNs acquire suppressive activity with increasing time after infection (21). Glycolysis is not impeded following *Hif1a* deletion, only its amplification, meaning that the basal glycolytic activity in effector leukocytes (i.e., monocytes and macrophages) would remain intact to exert antibacterial activity. Indeed, monocyte infiltrates were increased in *Mrp8^Cre^Hif1a^fl/fl^* mice with a concomitant decrease in bacterial burden. These findings supported the possibility that HIF1a could be amplifying glycolysis to a pathological point. To explore how HIF1a altered the transcriptional landscape of infiltrating leukocytes during PJI, scRNA-Seq was performed at early (day 3) and late (day 14) time points, which were selected since early metabolic intervention with 2-DG established durable phenotypic changes out to day 14 after infection. As predicted, G-MDSCs from *Mrp8^Cre^Hif1a^fl/fl^* mice were less glycolytic than their WT counterparts at both intervals; however, other pathways indicative of heightened proinflammatory activity were also evident. For example, *Mrp8^Cre^Hif1a^fl/fl^* G-MDSCs were enriched in cytoskeleton and leukocyte migration pathways during early infection (day 3), whereas, by day 14, IFN signaling, proinflammatory pathways, and oxidative phosphorylation were dramatically increased in various G-MDSC clusters from *Mrp8^Cre^Hif1a^fl/fl^* mice compared with WT littermates. Increased oxidative phosphorylation concomitant with reduced glycolysis is suggestive of metabolic reprogramming, which may partially explain why bacterial burden was reduced in *Mrp8^Cre^Hif1a^fl/fl^* animals. Importantly, targeting glycolysis using 2 independent approaches (i.e., 2-DG NPs and *Hif1a* conditional KO mice) resulted in similar outcomes, where granulocytes displayed heightened proinflammatory gene signatures coincident with marked decreases in bacterial abundance within the same tissue compartments, further strengthening the significance of our findings.

Examination of tissues from PJI patients revealed a similar increase in glycolysis and hypoxia pathways in tissue-infiltrating granulocytes compared with granulocytes from paired peripheral blood samples. These findings reinforce the glycolytic bias of granulocytes during PJI in humans, which is also associated with significant G-MDSC influx (22, 23). Other pathways of interest were IL-10 signaling, which was dramatically increased in granulocytes infiltrating infected tissues compared with blood. This is significant, since our prior work has established a key role for IL-10 in promoting PJI (17), in part, by the ability of *S*. *aureus* biofilm to produce lactate that leads to epigenetic reprogramming of leukocytes to favor IL-10 production (19). Conversely, several pathways were increased in peripheral blood granulocytes of PJI subjects, including WNT signaling, Rho and Cdc42 GTPases, and G protein–mediated events. By extension, these pathways were downregulated upon granulocyte extravasation from the vasculature into infected tissues; however, their significance on disease progression remains unknown. Interestingly, although 2 of the 3 patient samples were confirmed *S*. *aureus* PJI, there was no transcriptional footprint that separated these from the subject with a Gram-negative infection. This suggests a generalized response to PJI, although the small sample size makes a definitive conclusion speculative at the present time. Of note, the conserved glycolytic and hypoxia transcriptional signatures suggest they may be important nodes for regulating the host immune response to dictate PJI chronicity.

Despite our findings that the glycolysis/HIF1a axis is a key regulatory pathway for promoting G-MDSC antiinflammatory activity during PJI, there are several limitations of this study. The first relates to the inability to selectively regulate metabolic reprogramming in G-MDSCs versus PMNs based on the lack of unique surface markers for NP targeting or cell type–selective promoters for Cre-mediated gene excision. Nevertheless, since G-MDSCs dramatically outnumber PMNs at the site of PJI, this allowed interpretations of the data to be made. In addition, the identity of G-MDSCs versus PMNs could be deduced bioinformatically during scRNA-Seq, with expression profiles allowing for conclusions of metabolic biases and pro- versus antiinflammatory tendencies. Another limitation was the small number of human PJI samples for scRNA-Seq analysis, although all 3 subjects demonstrated a clear glycolytic/hypoxic bias, reinforcing observations in our mouse PJI model. To investigate the redox and metabolic properties of granulocytes when HIF1a signaling was perturbed in vitro, the small molecule inhibitor chetomin was used. The molecular target of chetomin is not HIF1a itself, but rather another subunit of the transcriptional complex, p300/CBP (47). Although chetomin has been widely used to attenuate HIF1a-mediated gene expression, it does raise the possibility that our in vitro data may be influenced by HIF1a-independent mechanisms, since p300/CBP is a translational coactivator for a variety of genes. An alternative explanation is that chetomin inhibited residual HIF1a activity in *Mrp8^Cre^Hif1a^fl/fl^* G-MDSCs. Nevertheless, our in vivo and sequencing data sets provide strong support for the role of Hif1a and genes under its control as important for the maladaptive immune response during chronic PJI.

The infection-associated changes in granulocyte metabolism following *S*. *aureus* PJI presented in this study are likely only part of a complex set of interacting factors. Although reducing glycolytic activity, either pharmacologically or genetically, was able to decrease bacterial burden and G-MDSC activity, this was not sufficient to clear infection. Therefore, it is likely that a combinational approach that pairs metabolic modulation with conventional antibiotics may prove more efficacious to combat PJI, a strategy that is supported by our prior work (16).

## Methods

### Sex as a biological variable.

Both male and female mice and human subjects were examined in this study, and similar findings are reported for both sexes.

### Mice.

C57BL/6J mice (RRID: IMSR_JAX:000664) were obtained from The Jackson Laboratory. Granulocyte *Hif1a* conditional KO mice (*Mrp8^Cre^Hif1a^fl/fl^*) were generated by crossing *Hif1a^fl/fl^* animals (RRID: IMSR_JAX:007561) with *Mrp8^Cre^*-GFP (RRID: IMSR_JAX:021614) mice, both from The Jackson Laboratory, with WT littermates (*Mrp8^Null^Hif1a^fl/fl^*) used as controls. All animals were bred in-house and mice of the same sex were randomly assigned into standard-density cages upon weaning (*n* = 5 per cage). Animals were housed in an access-restricted room equipped with ventilated microisolator cages, which were held at 21°C under 12-hour light/12-hour dark cycles. Mice were provided ad libitum access to water and chow (Teklad; Envigo), with Nestlets included in each cage for enrichment.

### S. aureus biofilm growth.

For each experiment, *S*. *aureus* USA300 LAC 13c (15) was grown on fresh blood agar plates from a glycerol stock. A single colony was inoculated into 25 mL of RPMI-1640 supplemented with 10% FBS, 1% HEPES, and 2 mM l-glutamine for overnight growth (16–18 hours) in a rotary shaker at 37°C, 250 rpm. A 1:100 dilution of the overnight culture (OD_600_ of 0.05) was used to inoculate 96-well plates that had been precoated with 20% human plasma to promote bacterial attachment as previously described (19). Biofilms were grown under static conditions at 37°C, and every 24 hours, approximately half of the medium was replenished, taking care to avoid disturbing the biofilm structure.

### Primary cell culture.

Primary G-MDSCs and macrophages were generated from BM cells isolated from the femurs and tibias of 8- to 10-week-old mice, as previously described (19). PMNs were recovered from the peritoneal cavity of mice 18 hours following an i.p. injection of 4% thioglycolate broth or the BM of naive mice using an anti-Ly6G microbead selection kit (catalog 130-120-337; Miltenyi Biotec). For coculture experiments, G-MDSCs or PMNs (2.5 × 10^5^ cells/well) were added to 4-day-old *S*. *aureus* biofilms and incubated at 37°C for 30 minutes. For experiments with 2-DG (catalog 14325; Cayman Chemical) or chetomin (catalog 14437; Cayman Chemical), granulocytes were pretreated for 1 hour prior to biofilm exposure with inhibitors present during the 30-minute coculture period.

### Measurement of glucose uptake, ROS, and H_2_O_2_.

After biofilm-granulocyte coculture, cells were washed and stained at 37°C in PBS + 10% FBS with 2-NBDG (catalog 11046; Cayman Chemical), Oxivision (catalog 11504; AAT Bioquest), MitoSOX (catalog M36008; Thermo Fisher Scientific), CellROX Green (catalog C10444; Thermo Fisher Scientific), or TMRM (catalog I34361; Thermo Fisher Scientific) for 30 minutes or MitoPY1 (catalog 4428; Tocris) for 60 minutes. Cells were analyzed using a LSRII (BD) or Attune Flow Cytometer (Thermo Fisher Scientific) with FlowJo software, version 10 (RRID:SCR_008520).

### Mouse model of S. aureus PJI.

To model infectious complications following prosthetic joint surgery, a mouse model of *S*. *aureus* PJI was used as previously described (16, 17, 21, 23, 24). Mice of both sexes between 8 and 10 weeks of age were utilized, and investigators were not blinded to treatment status or genetic background. Mice were anesthetized using ketamine/xylazine prior to an initial incision made through the quadriceps. A secondary incision was then created to laterally displace the patellar tendon prior to generating a burr hole in the distal end of the femur using a 26-gauge needle. An orthopedic-grade nickel-titanium Kirschner wire (0.6 mm diameter; Custom Wire Technologies) was inserted through the burr hole into the medullary canal, whereupon 10^3^ CFUs of *S*. *aureus* were inoculated at the tip of the wire. Slow-release Buprenex was administered subcutaneously following surgery to provide analgesia for 72 hours. Mice were monitored daily to ensure normal ambulatory function and the lack of discernible pain behaviors.

### Quantification of leukocyte infiltrates and bacterial burden.

The titanium implant, femur, knee joint, and surrounding soft tissue were collected from infected animals at designated time points following infection and processed as previously described (23). Serial 10-fold dilutions of tissue homogenates and supernatants from sonicated titanium implants were plated on TSA with 5% sheep blood, and bacterial burden was assessed the following day.

To quantify immune infiltrates within the soft tissue surrounding the infected joint, homogenates were filtered, and RBCs were eliminated by rapid lysis in H_2_O, with isotonic conditions returned with the addition of 10× PBS. Next, cells were incubated with TruStain FcX (catalog 101320; BioLegend) prior to staining with CD45-APC (RRID: AB_312977), Ly6G-PE (RRID: AB_1186099), CD11b-FITC (RRID: AB_312789), F4/80-PE-Cy7 (RRID: AB_893490), and Ly6C-PerCP-Cy 5.5 (catalog 560525; BD). Dead cells were excluded from analysis using a Zombie UV fixable Live/Dead stain (catalog 423108; BioLegend). All samples were analyzed on a BD LSR II (BD) with Spherotech AccuCount beads (catalog ACBP10010) to enable the reporting of absolute cell counts. All data were processed using FlowJo (RRID: SCR_008520) using the gating strategy presented in Supplemental Figure 11.

### Synthesis of 2-DG NP and in vivo delivery.

Boronic acid–modified copolymer was used as a sugar-trapping agent allowing 2-DG complex formation and delivery (58, 59). Freshly prepared complexes were stored at 4°C and used within 1 to 2 days. For 2-DG NP delivery, mice were placed in an induction chamber without restraint and anesthetized with 2.5% isoflurane. A single dose of 2-DG–containing (25 μL of 3.5 mg/mL 2-DG in 1× PBS) or empty NPs was administered by injection into the soft tissue surrounding the infected joint on day 3 after infection. In some experiments, a Cy5-containing NP was administered to monitor leukocyte uptake during the first 3 days after injection by flow cytometry.

### RNA-Seq.

For scRNA-Seq analysis of leukocytes at various stages of PJI, live CD45^+^ cells were collected from the soft tissue adjacent to the knee joint of C57BL/6J mice at days 3, 7, and 14 after infection by FACS and prepared for sequencing as previously described (19). For analysis of *Mrp8^Cre^Hif1a^fl/fl^* mice, live CD45^+^ cells were collected from the joint tissue of *Mrp8^Cre^Hif1a^fl/fl^* and WT littermates (*Mrp8^Null^Hif1a^fl/fl^*) at days 3 and 14 after infection by FACS.

To explore the leukocyte transcriptional landscape during human PJI, scRNA-Seq was performed on tissue samples acquired from patients undergoing revision arthroplasty due to infection (Supplemental Table 1). Blood was also obtained from each subject as a comparator with tissue-infiltrating leukocytes. Single-cell suspensions were prepared and analyzed for viability, but were not sorted to minimize cell death because of the transit time from the operating room to the laboratory. The infectious agent for each subject was identified by the Clinical Microbiology Laboratory at UNMC and, due to the conditions of the IRB protocol, background information for each patient, such as length of infection, comorbidities, and antibiotic use, was not available.

Prior to single-cell capture, cell suspensions were evaluated using a Luna automated fluorescent cell counter (Logos Biosystems) to assess sample density, viability, and presence of debris. Samples were loaded onto a 10X Genomics instrument, and single cells were captured and lysed; cellular RNA was reverse transcribed and barcoded using a Chromium Single Cell 3′ Reagent Kit (version 3.1; 10X Genomics) according to the manufacturer’s instructions. Illumina-compatible cDNA libraries were created and quantified using a Qubit-30 Fluorometer and evaluated with a fragment analyzer. The libraries were loaded on a Novaseq6000 instrument at a final concentration of 300 pM. The samples were sequenced with parameters suggested by 10X Genomics to an average depth of 50,000–100,000 reads per cell.

To determine how 2-DG NPs influenced granulocyte transcriptional profiles, viable CD45^+^Ly6G^+^ cells were purified from the soft tissue surrounding the infected joints of C57BL/6J mice by FACS at day 7 after infection (4 days following 2-DG NP injections) for bulk RNA-Seq. RNA was immediately isolated from purified cells using a Quick-RNA MicroPrep Kit (catalog R1050; Zymo Research) according to the manufacturer’s instructions. Libraries were constructed with 10 ng of total RNA per sample that was reverse transcribed using the Smarter V4 Ultra Low Input RNA Kit (catalog 634888; Takara) with 7 cycles of PCR amplification to generate cDNA. The cDNAs (1 ng per sample) were converted to Illumina sequencing libraries using the Nextera XT Kit (catalog FC-131-1024; Illumina). Libraries were sequenced following a 2 × 75 bp paired-end strategy on a NovaSeq 6000 sequencer to generate approximately 120 million pairs of reads per sample.

### Bioinformatics.

Single-cell expression data were mainly analyzed using the tools provided in the Seurat package (60). Raw sequencing reads were preprocessed using the 10X Genomics Cell Ranger Pipeline, and the resultant matrices were imported into an R environment as Seurat objects with cells expressing at least 200 features across a minimum of 3 cells. A standard quality control procedure was implemented to safeguard against contamination by low-quality cells/reads. Low-quality cells were defined as those with too few (<200) or too many (>2,500) features and/or excessive (>5% of counts) mitochondrial contamination. All remaining cells and features were used for downstream analyses. Cell-type identities were annotated using the SingleR and celldex R packages (61). Gene counts were log-normalized prior to comparison against a reference expression set from the Immunological Genome Project (ImmGen; https://www.immgen.org/). Erroneous and ambiguous annotation results were removed using an outlier-based approach as implemented in SingleR. Each individual sample was then independently normalized using a regularized negative binomial regression model as implemented in the sctransform method (62, 63), which is a part of the Seurat package. Each object was then combined into a single object using a standard pipeline for iterative pairwise integration of Seurat objects (64). The dimensionality of the data was then reduced using PCA and UMAP approaches, and the first 30 components were used for course-grained (resolution = 0.5) clustering.

Differential expression testing, within and between clusters, was conducted using the MAST approach as implemented within the Seurat framework (65). For within-cluster comparisons, the clusters were split based on differentiating characteristics (i.e., genotype), normalized, scaled, and compared against each other on a cluster-by-cluster basis. The resultant differential expression data were then used for downstream pathway analyses using the fgsea (66) and MSigDB (67) R packages. The Hallmark, canonical, and gene ontology sets were chosen as references depending on the desired level of granularity required for analysis. Data for bulk RNA-Seq of Ly6G^+^ granulocytes recovered from mice receiving 2-DG versus empty NPs were analyzed using the Partek Flow Genomics Suite (RRID: SCR_011860). Quality check and trimming were performed before alignment, with additional quality steps executed after alignment to remove features with low read counts. The data were normalized by counts per million, and differentially expressed genes were identified using DESeq2 (68). Genes with *P* < 0.05 were used to identify differentially regulated pathways within Partek Flow.

Metabolic modeling was performed using the COMPASS algorithm, which allows for the derivation of metabolic subsystem activities using single-cell expression data as a set of constraints during flux balance analysis with genome-scale metabolic models (38). To reduce computational overhead during modeling, a subset of Seurat object read counts was used. For G-MDSC versus PMN comparisons in C57BL/6J mice, the first 2 clusters (G1, G2) were selected as representative G-MDSCs and the final 2 clusters (G9, G10) as PMNs, which was based on the expression of canonical gene sets reported for each cell type (37). In analyses that focused on the impact of *Hif1a*, the first 3 clusters (G1, G2, G3) were selected and split according to genotype (i.e., *Mrp8^Null^* or *Mrp8^Cre^*) prior to analysis. To further reduce computational overhead, each representative sample was compressed to approximately 100 representative microclusters. Each sample was then independently analyzed using the default parameters except for sample species, which was changed to *Mus musculus*. Resultant reaction penalties were analyzed in a Python environment. Postprocessing was performed in a manner similar to that used in the original COMPASS report (38). Briefly, penalties were converted into scores by taking the negative logarithm prior to grouping reactions into metareactions using a hierarchical clustering approach. Samples were then compared using Wilcoxon’s rank-sum test with the goal of identifying metabolic pathways that were enriched in either subset. The results were then filtered according to their reaction confidence scores (4, high, or 0, unassigned) in the reference metabolic model. Downstream analyses and figure generation were performed using the filtered data in an R environment using custom scripts that have been deposited in GitHub (https://github.com/KielianLab/2024_Horn-Arumugam).

### Statistics.

Significant differences were determined by an unpaired, 2-tailed Student’s *t* test or 1- or 2-way ANOVA with Tukey’s correction, as described in the figure legends, using GraphPad Prism (RRID: SCR_002798), where *P* < 0.05 was considered statistically significant.

### Study approval.

Animal studies were conducted with strict adherence to recommendations in the *Guide for the Care and Use of Laboratory Animals* (National Academies Press, 2011). The animal use protocol was approved by the UNMC IACUC (no. 18-013-03). Informed consent for procuring tissue samples from human PJI subjects was obtained under a protocol approved by the UNMC Institutional Review Board (no. 0657-13).

### Data availability.

The data underlying Figure 3, Figure 4, B–H, Figure 5, A–C, Figure, 6, Figure 7, and Figure 8 are available in the published article. All RNA-Seq data sets described in this study and underlying Figure 1, Figure 2, Figure 4A, Figure 5, D–G, Figures 9, Figure 10, and Figure 11 are available in the NCBI’s Gene Expression Omnibus database (GEO GSE242039). Values for all data points in graphs are reported in the Supporting Data Values file.

## Author contributions

CMH, PA, CEH, ZVR, and TK designed experiments. CMH, PA, CEH, BPB, and ZVR conducted experiments. DS and VCT performed metabolomics analysis. SGR and TKB conceptualized and synthesized the 2-DG NP. RWF generated the *Mrp8^Cre^Hif1a^fl/fl^* conditional KO mice and performed genotyping and husbandry. KLG and CWH provided surgical specimens from PJI patients. CMH wrote the initial manuscript draft, with PA responsible for the revision. Co–first authorship order was based on timeline for project involvement. All authors edited and approved the final manuscript.

## Supplementary Material

Supplemental data

Unedited blot and gel images

Supporting data values

## Version 1 02/29/2024

In-Press Preview

## Version 2 04/15/2024

Electronic publication
