# Supplementary material for: Granulocytic myeloid-derived suppressor cell activity during biofilm infection is regulated by a glycolysis/HIF1a axis
Source: J Clin Invest. 2024 Feb 29;134(8):e174051. doi: 10.1172/JCI174051 (PMC11014666; doi:10.1172/JCI174051)
Supplement: Supplemental data [file jci-134-174051-s012.pdf]

SUPPLEMENTAL METHODS

Synthesis of 2-DG nanopreparation (NP). 3-aminophenylboronic acid (Cat. #900988), *N*-hydroxysuccinimide (NHS; Cat. #130672), and 1-(3-dimethylaminopropyl)-3-ethyl carbodiimide hydrochloride (EDC; Cat. #341006) were purchased from Sigma Aldrich (St. Louis, MO). All reagent-grade chemicals purchased from Sigma-Aldrich were used without any purification. Poly (ethylene glycol)-b-poly(L-glutamic acid) (PEG-b-*p*Glu) block copolymer ($Đ_M$ = 1.08) was synthesized as previously described (1). The block lengths were 114 and 150 repeating units for PEG and *p*Glu, respectively. 3-aminophenylboronic acid (BA) was conjugated to PEG-*p*Glu block copolymer via a coupling reaction between the carboxyl group of *p*Glu and amino group of BA in the presence of EDC and NHS. The activation reaction (EDC/NHS) was conducted in water upon stirring for 5 min followed by addition of 3-aminophenylboronic acid (5 eq. with respect to Glu units). After stirring for 24 h at room temperature, the reaction mixture was purified by dialysis against deionized water for 48 h using a 3,500 Da cutoff membrane. The resulting PEG-*p*(Glu-*g*-BA) copolymer was freeze-dried, and its functionalization with BA and purity were confirmed by ^{1}H-NMR spectra recorded in D_2O at 25°C using a Bruker 400 MHz spectrometer. Next, 2-DG-polymer complexes were prepared by gentle mixing of appropriate amounts of stock solutions of PEG-*p*(Glu-*g*-BA) (10 mg/mL in 50 mM phosphate buffer, pH 7.4) and 2-DG (10 mg/mL in distilled water, 5 eq. with respect to BA units) followed by stirring for 24 h at room temperature. Excess 2-DG was removed using Amicon filter units (MWCO 3,500 Da). The amount of free (non-formulated) drug was estimated using a sugar assay (2). Freshly prepared complexes were stored at 4°C and used within 1-2 days.

Western blot and RT-qPCR. To validate effective *Hif1a* excision in G-MDSCs from *Mrp8^{Cre}Hif1a$^{fl/fl}$* mice, expression of *Hif1a* using primers spanning the deletion site (exons 2 – 3) and several Hif1a-dependent genes was determined by RT-qPCR. G-MDSCs or macrophages (as a control) were cultured for 16 h under normoxia or hypoxia (1% O_2) to augment HIF1a action, whereupon total RNA was isolated using TRIzol reagent (Cat. #15596018; Thermo Fisher Scientific) and genomic DNA removed using DNase I (Cat. #AMPD1-1KT, Sigma). RNA was used to generate cDNA using a Verso cDNA synthesis kit (Cat. #AB1453B; Thermo Fisher Scientific) and qPCR was performed with TaqMan probes [*Hif1a* (Cat. #Mm01283757_m1), hexokinase 2 (*Hk2*; Cat. #Mm00443385_m1), glutamate transporter 1 (*Glut1*; Cat. #Mm00441480_m1), vascular endothelial growth factor alpha (*Vegfa*; Cat. #Mm00437306_m1), and β-actin (*Actb*; Cat. #Mm02619580_g1)] (all from Thermo Fisher Scientific) using a CFX Connect Real-Time system (Bio-Rad). Data were normalized to β-actin and are

reported as relative expression ($2^{-\Delta\Delta Ct}$, where Ct is the cycle threshold) compared to normoxia samples for each genotype.

To examine HIF1a protein expression in G-MDSCs and macrophages from *Mrp8^{Cre}Hif1a$^{fl/fl}$* vs. WT littermates (*Mrp8^{Null}Hif1a$^{fl/fl}$*), cells were treated with cobalt chloride (500 µM) for 24 h, lysed directly in Laemmli buffer (Cat. #161-0737; Bio-Rad) and incubated at 95°C for 10 min. Proteins were separated using 10% SDS-polyacrylamide gel electrophoresis, transferred to a polyvinylidene fluoride membrane (Cat. #ISEQ00010; Merck Millipore) and incubated overnight with a rabbit HIF1a mAb (RRID:AB_2799095). The following day, the membrane was washed and incubated with an anti-rabbit IgG-HRP conjugated secondary Ab (RRID:AB_2099233) for 1 h at room temperature and developed using Clarity Western ECL substrate (Cat. #170-5060; Bio-Rad) for detection in a Western blot imaging system (Azure 600; Azure Biosystems). Blots were stripped and re-probed with a β-actin-HRP conjugated Ab (RRID:AB_867494) as a loading control.

Effects of 2-DG and chetomin on bacterial growth and metabolism. *S. aureus* was cultured under planktonic or biofilm conditions with a concentration of 2-DG equivalent to the entire drug content delivered by NPs in vivo (~4.5 µg) or 375 nM chetomin that was used for in vitro biofilm-leukocyte co-cultures. For planktonic culture, drugs were added at time 0, and bacterial growth was measured at 600 nm over a 24 h period in a microplate reader (Tecan). For biofilm culture, 2-DG or chetomin was included during the initiation of biofilm formation and daily medium exchanges. In separate experiments, mature biofilm was treated with chetomin for 30 min to reflect the interval that biofilms were exposed to the compound during biofilm-leukocyte co-culture studies. Samples were serially diluted and plated to assess the number of viable bacteria reported as CFU.

To assess the effects of 2-DG on *S. aureus* metabolism, planktonic or biofilm cultures were collected at the indicated time points and washed twice with a 0.6% NaCl solution. The pellet was resuspended in 60% EtOH, and cells were lysed using a Precellys homogenizer for 30 sec at 6,800 rpm. The lysate was centrifuged at 12,000 rpm for 5 min, whereupon the supernatant containing metabolites was collected and lyophilized. Lyophilized samples were resuspended in 100 µl of 50% MetOH and 80 µl of each sample was loaded for targeted LC-MS/MS. Detection and quantification of polar metabolites was performed using a Triple-quadrupole-ion trap hybrid mass spectrometer (QTRAP6500+; Sciex, USA) connected with an ultra-performance liquid chromatography (UPLC) *I*-class system (Waters, USA). The chromatographic separation of

metabolites was performed using a HILIC XBridge Amide analytical column (150 mm x 2.1 mm i.d.; particle size 1.7 µm; Waters, USA) and a binary solvent system in gradient mode. Mobile phase A was composed of ammonium acetate and ammonium hydroxide (10 mM each) containing 5% acetonitrile in LC-MS grade water. The pH was adjusted to 8.0 using glacial acetic acid and mobile phase B was 100% LC-MS grade acetonitrile. The column was maintained at 40°C at a flow rate of 0.3 mL/min, and the autosampler temperature was held at 5°C.

The QTRAP6500+ was operated in negative and positive polarity switching mode for targeted analysis of metabolites by Multiple Reaction Monitoring (MRM) using previously optimized LC-MS/MS parameters (3). Q1/Q3 pairs for metabolite detection are provided in **Supplemental Table 2**. $^{13}C^{15}N$-labeled canonical amino acid mix (Cat. #MSK-CAA-1; Cambridge Isotope Laboratory) was used as an internal standard. Analyst and Multiquant software (Sciex, USA) was employed for data acquisition and processing, respectively.

SUPPLEMENTAL REFERENCES

1. Desale SS, Cohen SM, Zhao Y, Kabanov AV, and Bronich TK. Biodegradable hybrid polymer micelles for combination drug therapy in ovarian cancer. *J Control Release.* 2013;171(3):339-48.
2. Masuko T, Minami A, Iwasaki N, Majima T, Nishimura S, and Lee YC. Carbohydrate analysis by a phenol-sulfuric acid method in microplate format. *Anal Biochem.* 2005;339(1):69-72.
3. Bulock LL, Ahn J, Shinde D, Pandey S, Sarmiento C, Thomas VC, et al. Interplay of CodY and CcpA in Regulating Central Metabolism and Biofilm Formation in Staphylococcus aureus. *J Bacteriol.* 2022;204(7):e0061721.

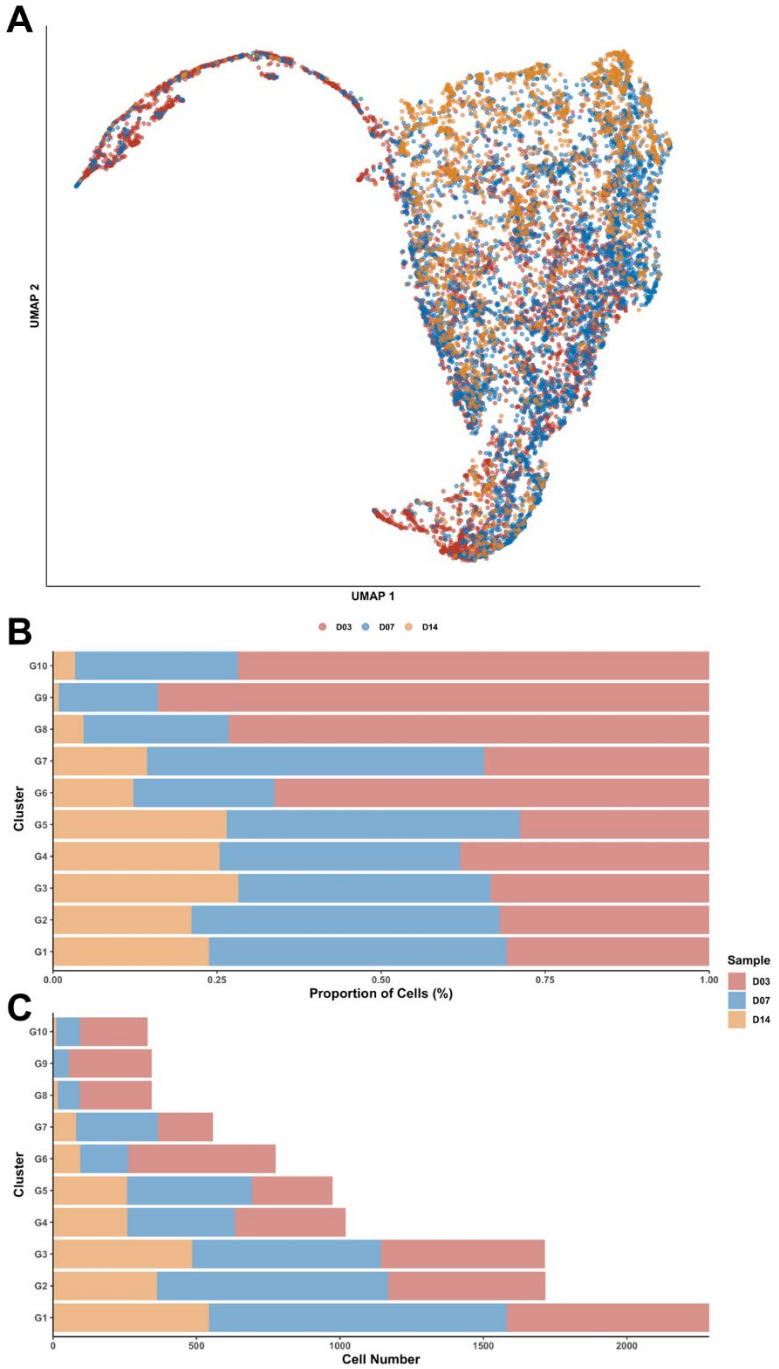

Supplemental Figure 1. Cluster composition of granulocytes recovered from the mouse PJI model for scRNA-seq. CD45^{+} leukocytes were isolated from the soft tissue surrounding the infected joint of mice at days 3, 7, and 14 post-infection for scRNA-seq. Data was integrated to generate (**A**) UMAP clustering of granulocytes, colored by day post-infection. (**B**) Proportions of granulocyte clusters from each sample origin and (**C**) discrete counts of granulocytes within each cluster, colored by day post-infection.

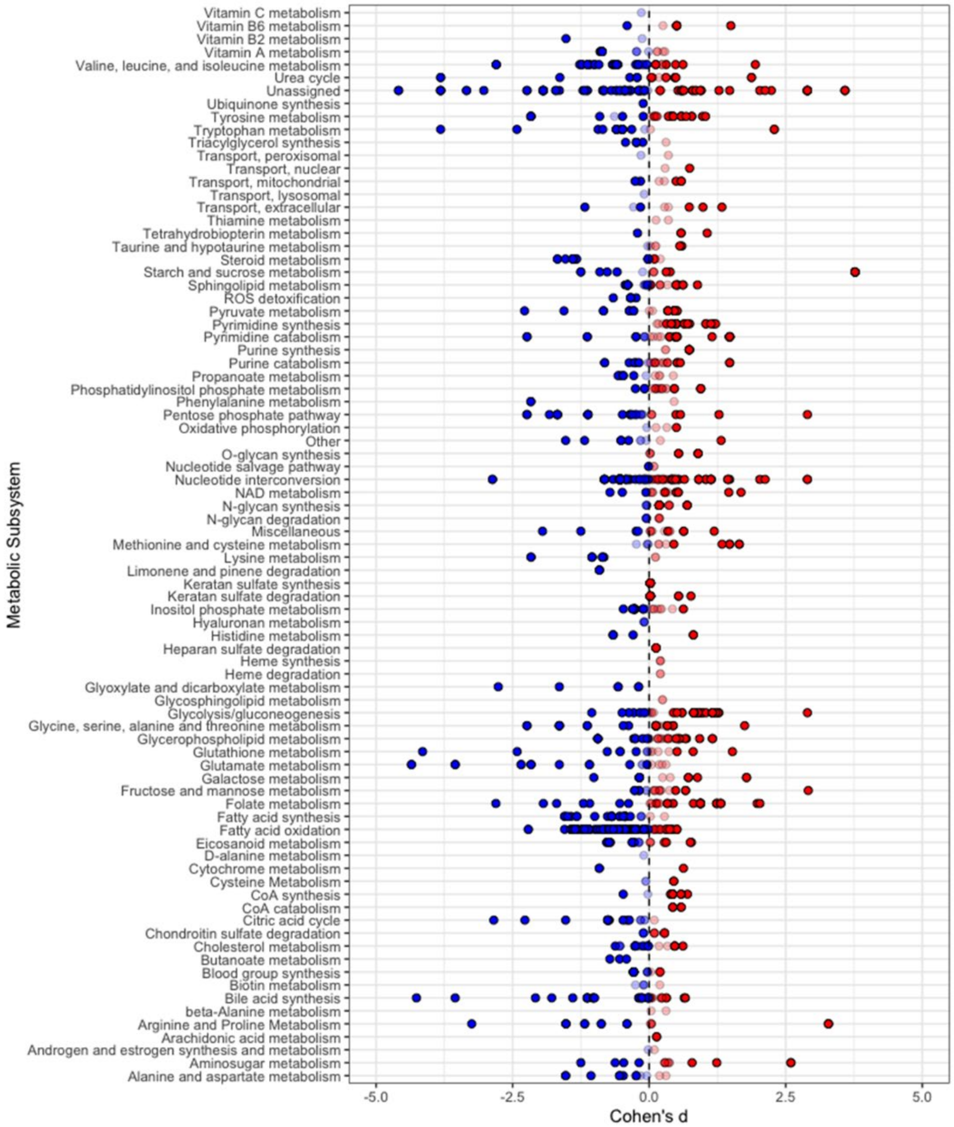

Supplemental Figure 2. G-MDSCs and PMNs exhibit distinct metabolic biases. Compass scores of G-MDSCs and PMNs from the integrated scRNA-seq dataset at days 3, 7, and 14 post-infection were used to generate Cohen's D to reflect the relative metabolic tendencies of each cell type. Each metabolic reaction was grouped into metabolic subsystems and plotted (red: G-MDSC; blue: PMN).

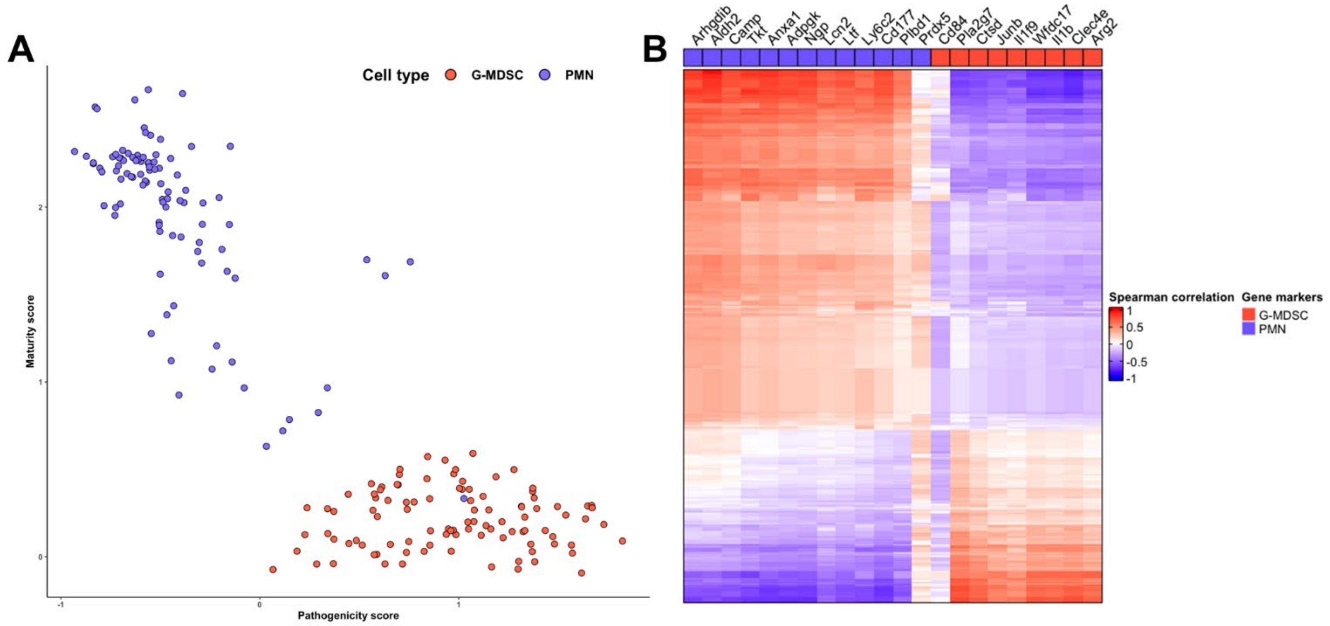

Supplemental Figure 3. Metabolic activity is strongly linked to cellular identity. Compass analysis was performed using the integrated scRNA-seq dataset at days 3, 7, and 14 post-infection to (**A**) score granulocytes on expression of G-MDSC (pathogenicity) or PMN (maturity) gene sets. (**B**) Correlation of metabolic activity, as predicted by Compass, and the expression of genes in the G-MDSC and PMN sets used for separation of the populations in (**A**).

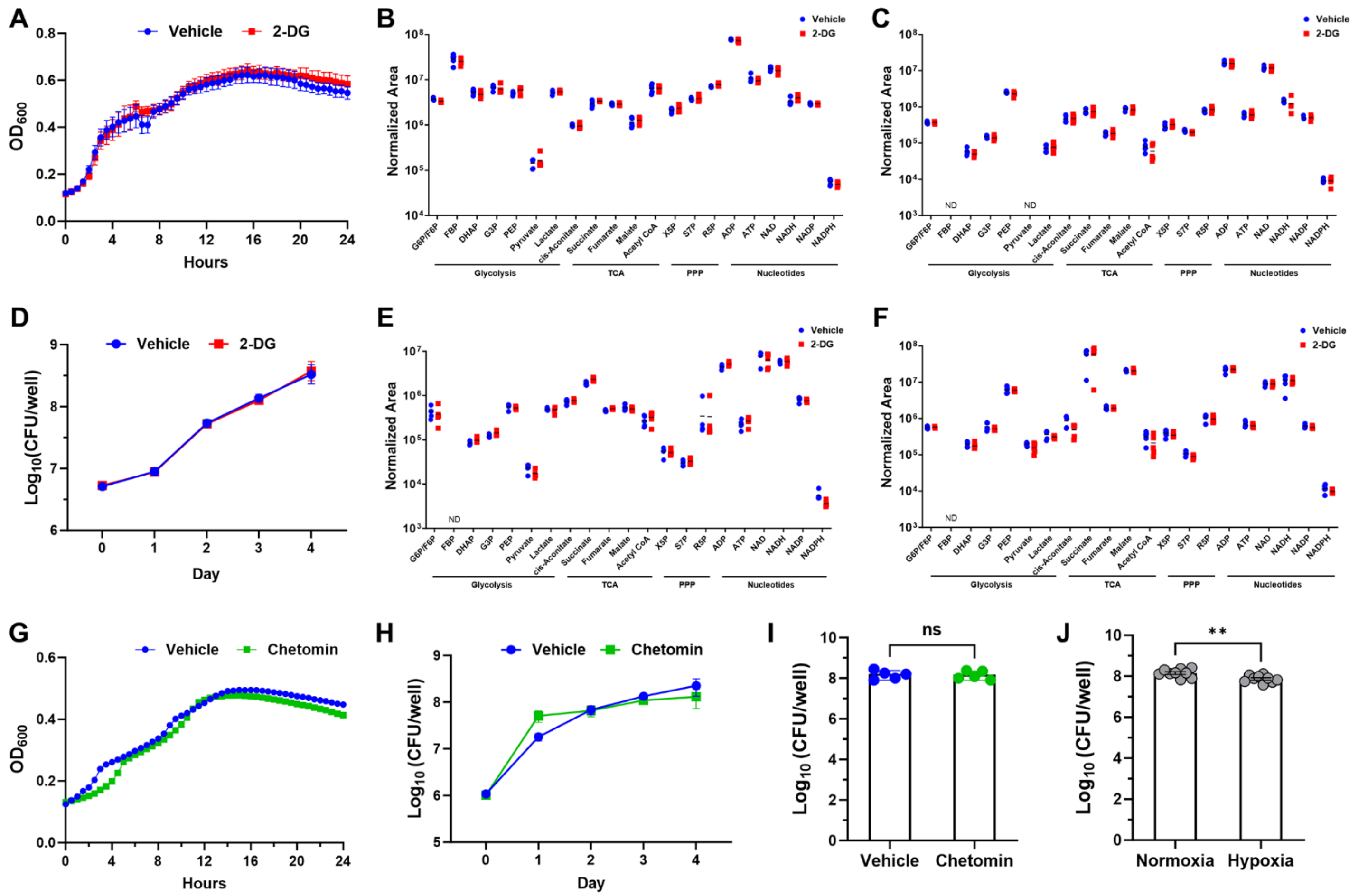

Supplemental Figure 4. 2-DG and chetomin do not affect *S. aureus* growth or metabolism. *S. aureus* growth following treatment with 4.5 µg/ml 2-DG or vehicle during (**A**) planktonic culture at time 0 and (**B-C**) intracellular metabolites were quantified by targeted LC-MS/MS during planktonic growth at (**B**) 6 h or (**C**) 24 h (data represent mean ± SEM; n = 5/group). (**D**) *S. aureus* biofilm growth following exposure to 4.5 µg/ml 2-DG or vehicle at the initiation of biofilm culture (day 0) and throughout the 4-day maturation period (data represent mean ± SEM; n = 15 /group). Intracellular metabolites of (**E**) *S. aureus* biofilm treated with 4.5 µg/ml 2-DG or vehicle at the initiation of biofilm growth with metabolites quantified at 24 h and (**F**) a mature 4-day old biofilm treated with 4.5 µg/ml 2-DG for 3 days (n = 5/group). Biofilm cultures were replenished daily with fresh medium containing 4.5 µg/ml 2-DG. (**G-I**) *S. aureus* was treated with 375 nM chetomin during (**G**) planktonic growth (data represent mean ± SEM; n = 24/group), (**H**) the initiation of biofilm formation from day 0 to day 4 (biofilm medium was replaced daily with fresh media containing chetomin; data represent mean ± SD; n = 5/group; chetomin day 4, n = 4), or (**I**) a 4-day mature biofilm treated for 30 min (data represent mean ± SD; n = 5/group). (**J**) Bacterial titers in *S. aureus* biofilms grown for a 4-day period under normoxia or hypoxia are expressed as colony forming units (CFU)/well (data represent mean ± SEM; n = 9/group; **, $p < 0.01$; unpaired two-tailed t-test).

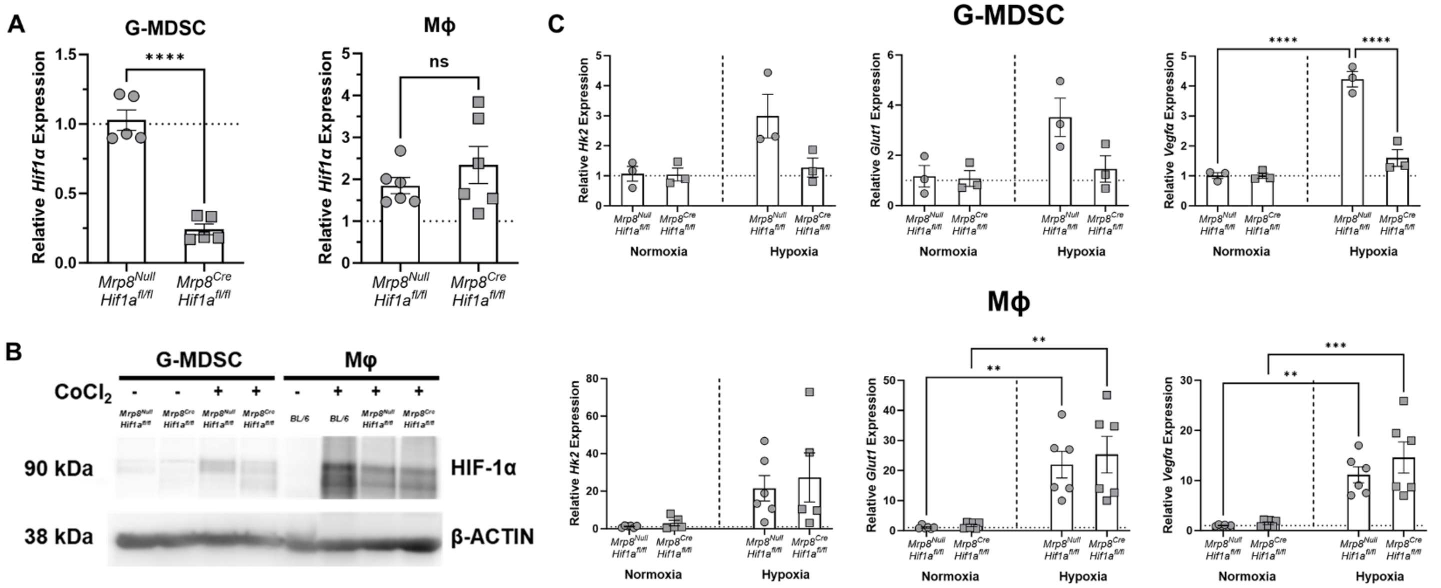

Supplemental Figure 5. Conditional *Hif1a* deletion in granulocytes prevents the induction of Hif1a-depenent genes in G-MDSCs, but not macrophages (Mφs), in response to hypoxia. (**A**) *Hif1a* mRNA levels in bone marrow-derived G-MDSCs and Mφs from *Mrp8^{Null}Hif1a$^{fl/fl}$* and *Mrp8^{Cre}Hif1a$^{fl/fl}$* mice (G-MDSC, n = 5/group; Mφ, n = 6/group; ****, $p < 0.0001$; unpaired two-tailed t-test) and (**B**) HIF1a protein expression in G-MDSCs and Mφs from *Mrp8^{Null}Hif1a$^{fl/fl}$* and *Mrp8^{Cre}Hif1a$^{fl/fl}$* mice treated with 500 μM cobalt chloride ($CoCl_2$) for 24 h. (**C**) G-MDSCs and Mφs from *Mrp8^{Null}Hif1a$^{fl/fl}$* and *Mrp8^{Cre}Hif1a$^{fl/fl}$* mice were cultured in normoxia or hypoxia (1% O_2) for 16 h, whereupon *Hif1a*-dependent genes were quantified by RT-qPCR (G-MDSC, n=3/group; Mφ, *Hk2*, n = 6 for *Mrp8^{Null}Hif1a$^{fl/fl}$* and n = 5 for *Mrp8^{Cre}Hif1a$^{fl/fl}$*; *Glut1* and *Vegfa*, n = 6/group; **, $p < 0.01$; *** $p < 0.001$; **** $p < 0.0001$; one-way ANOVA with Tukey's correction). Data represent mean ± SEM.

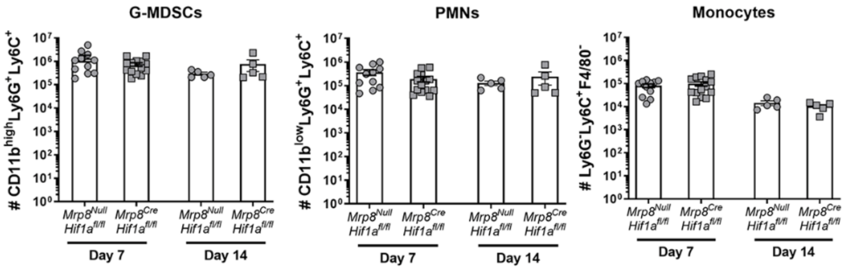

Supplemental Figure 6. Absolute numbers of leukocyte infiltrates in WT and *Mrp8Hif1a* conditional knockout mice. $Mrp8^{Null}Hif1a^{fl/fl}$ and $Mrp8^{Cre}Hif1a^{fl/fl}$ mice were euthanized at days 7 and 14 following *S. aureus* PJI, whereupon the absolute numbers of G-MDSC, PMN, and monocyte infiltrates were determined by flow cytometry using counting beads (data represent mean ± SEM; day 7, n = 11 for $Mrp8^{Null}Hif1a^{fl/fl}$ and n = 14 for $Mrp8^{Cre}Hif1a^{fl/fl}$; day 14, n = 5/group).

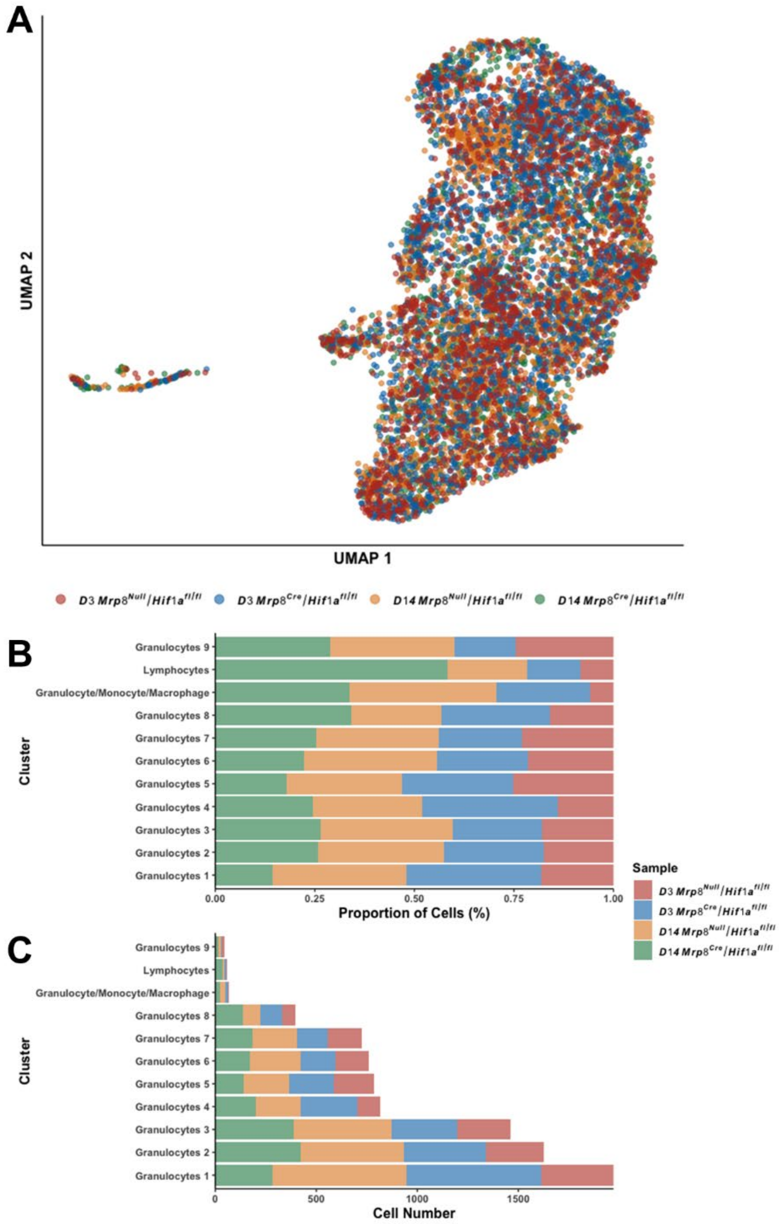

Supplemental Figure 7. Cluster composition of granulocytes recovered from *Mrp8^{Cre}Hif1a$^{fl/fl}$* mice for scRNA-seq. CD45^{+} leukocytes were isolated from the soft tissue surrounding the infected joint of *Mrp8^{Null}Hif1a$^{fl/fl}$* and *Mrp8^{Cre}Hif1a$^{fl/fl}$* mice at days 3 and 14 post-infection for scRNA-seq. Data was integrated to generate (**A**) UMAP clustering of granulocytes, colored by sample identity. (**B**) Proportions of granulocyte clusters for each sample origin and (**C**) absolute counts of granulocytes within each cluster, colored by sample identity.

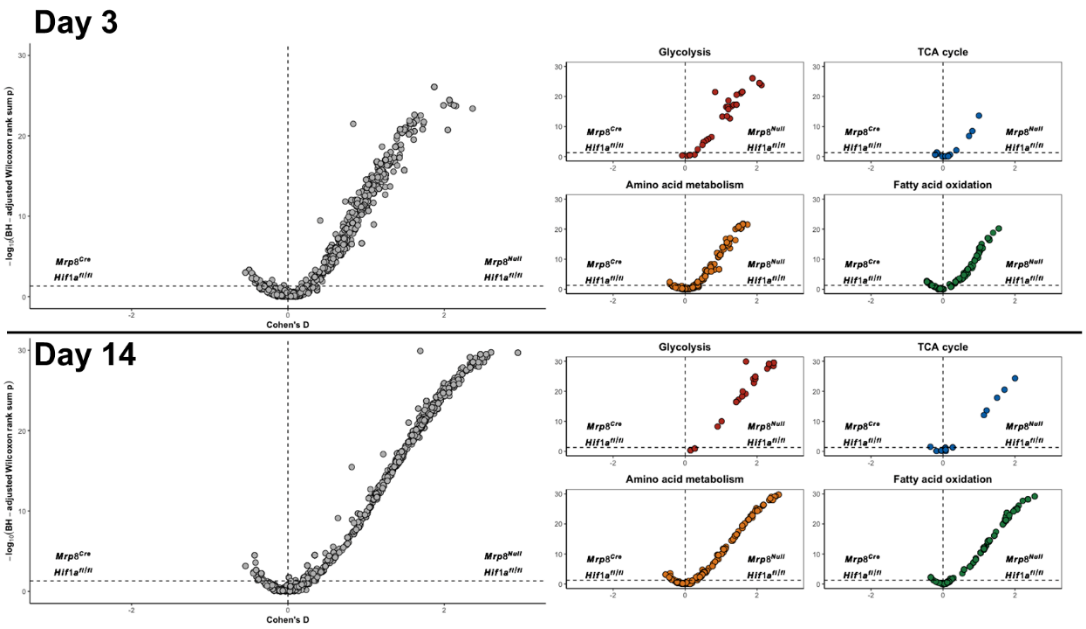

Supplemental Figure 8. Conditional knockout of *Hif1a* in granulocytes causes substantial decreases in the predicted activity of nearly all metabolic subsystems. scRNA-seq data from *Mrp8^{Null}Hif1a$^{fl/fl}$* and *Mrp8^{Cre}Hif1a$^{fl/fl}$* granulocytes were analyzed using Compass to investigate potential metabolic differences between the groups. The resultant score matrices for each group at days 3 and 14 post-infection were compared to yield predicted relative activities.

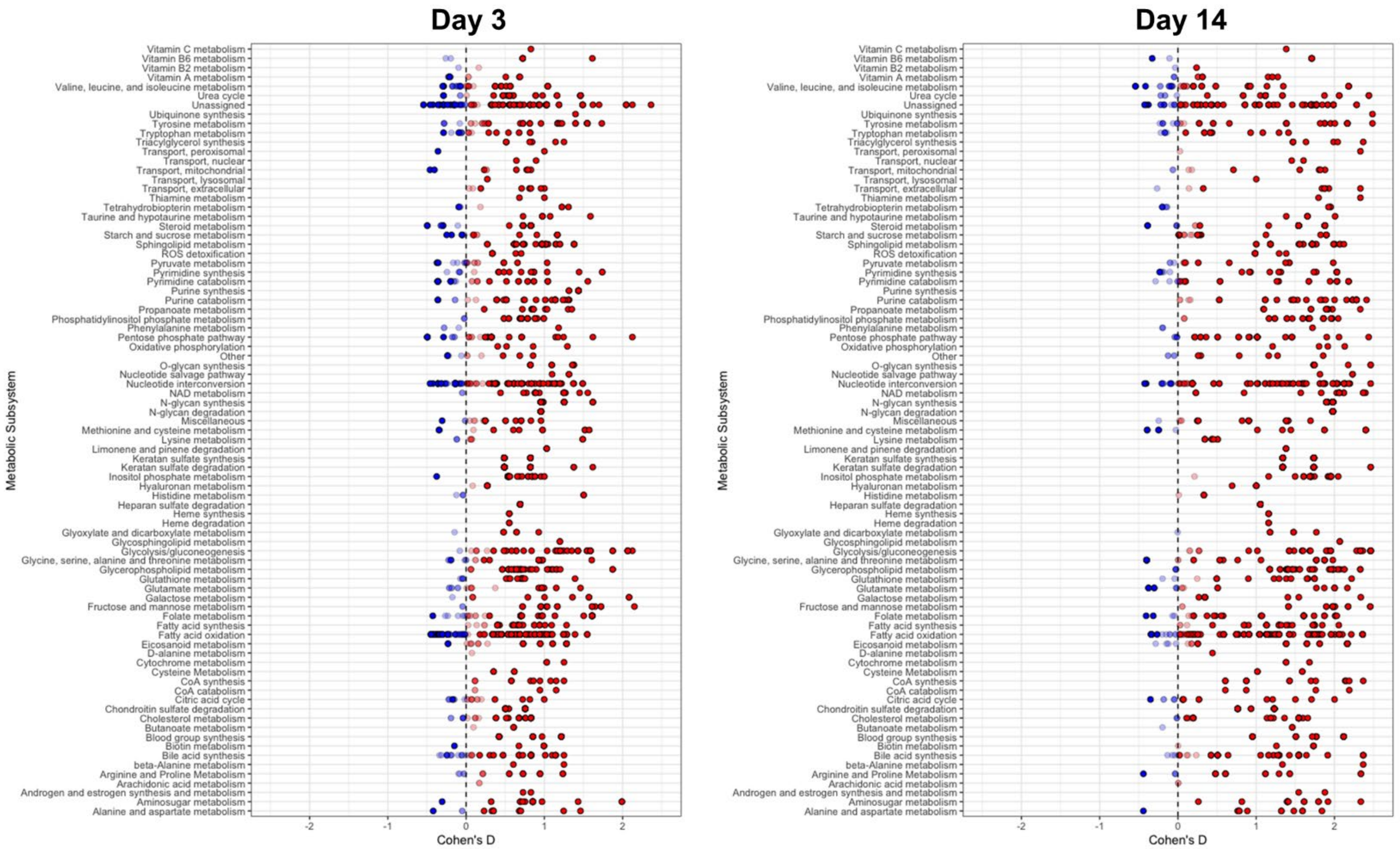

Supplemental Figure 9. Granulocytes isolated from *Mrp8^{Cre}Hif1a$^{fl/fl}$* mice following *S. aureus* PJI display major metabolic changes. Compass scores of granulocytes from *Mrp8^{Null}Hif1a$^{fl/fl}$* (red) and *Mrp8^{Cre}Hif1a$^{fl/fl}$* (blue) mice at days 3 and 14 post-infection from the scRNA-seq dataset were used to generate Cohen's D to reflect the relative metabolic tendencies of each genotype. Individual metabolic reactions were grouped into metabolic subsystems and plotted.

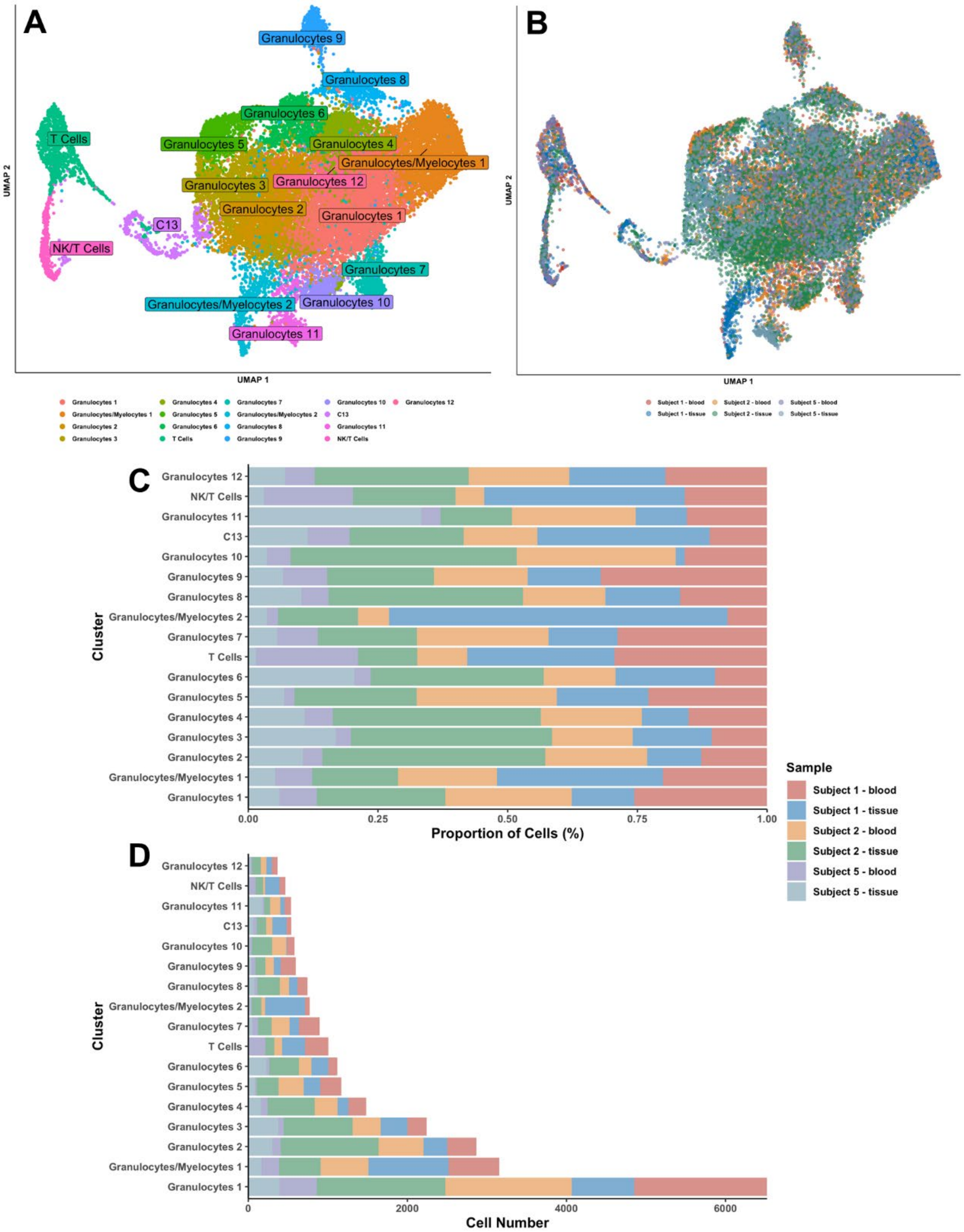

Supplemental Figure 10. Cluster composition of scRNA-seq from prosthetic joint infection (PJI) subjects. Matched tissue and blood samples from PJI patients were processed by scRNA-seq and data integrated. UMAP clustering, colored by (**A**) clusters and (**B**) sample origin. The (**C**) proportions and (**D**) cell counts within each cluster, colored by sample origin are shown.

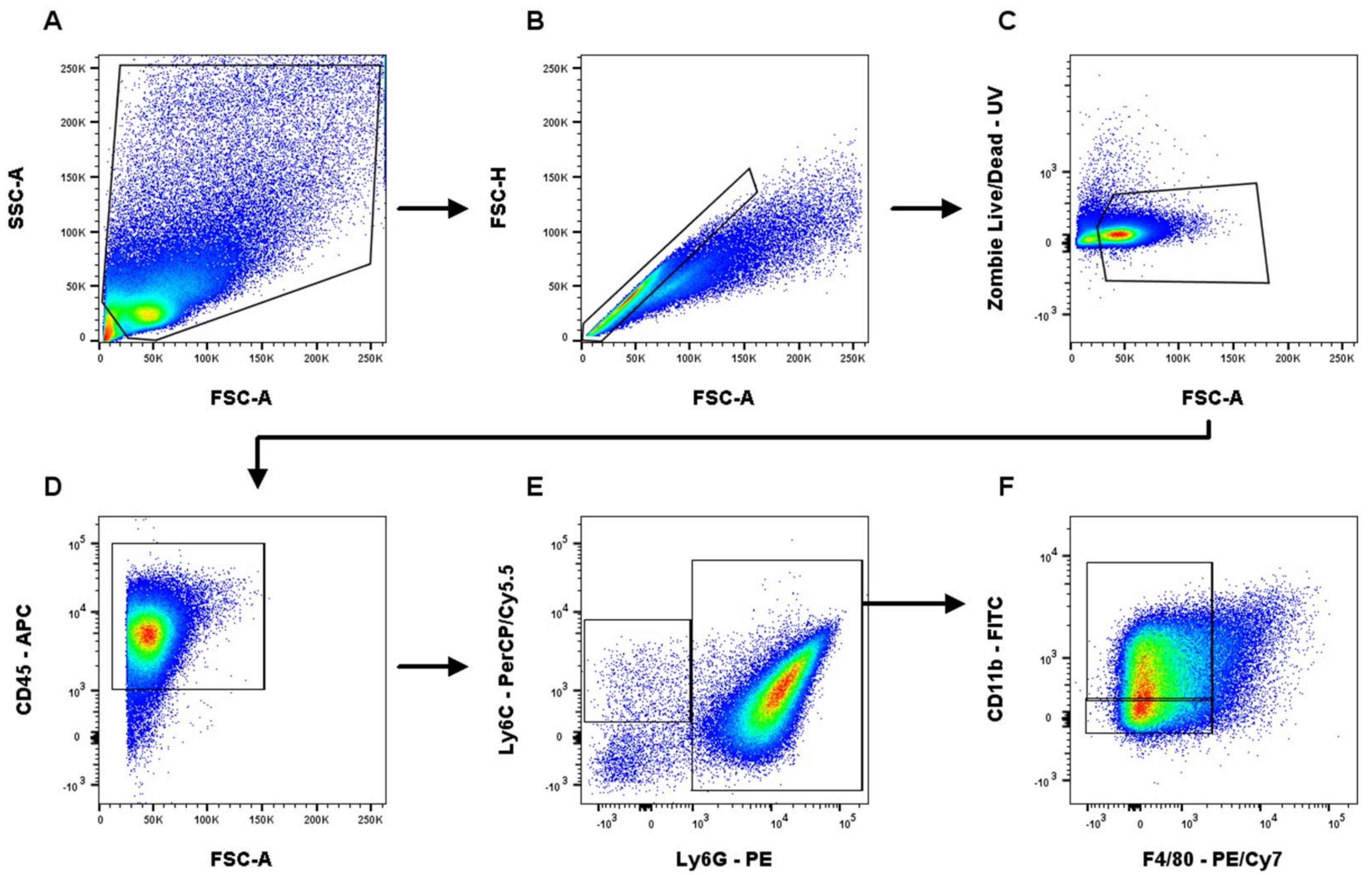

Supplemental Figure 11. Flow cytometry gating strategy for quantifying leukocyte infiltrates during *S. aureus* PJI. From the (**A**) total population, (**B**) single cells were gated using FSC-A vs. FSC-H prior to (**C**) excluding dead cells. (**D**) Live CD45$^+$ leukocytes were divided based on their expression of (**E**) Ly6C and Ly6G into Ly6G$^-$Ly6C$^+$ monocyte and Ly6G$^+$Ly6C$^+$ populations, the latter of which was characterized as (**F**) PMNs and G-MDSCs based on CD11b expression.

Supplemental Table 1. Demographics of human PJI subjects.

Subject #	Sex	Age	Race	Infectious diagnosis
1	F	47	Caucasian	*Staphylococcus aureus*
2	M	74	Caucasian	*Stenotrophomonas maltophilia*
5	M	58	Caucasian	*Staphylococcus aureus*

Supplemental Table 2. Multiple Reaction Monitoring details for metabolites

Component Name	Mass Info (Q1/Q3)
Acetyl CoA	810.2 / 303.2
ADP	428.0 / 136.0
ATP	508.0 / 410.0
cis-Aconitate	173.0 / 129.0
D-Fructose 1,6-bisphosphate	339.0 / 79.0
D-Fructose 1/6-phosphate	259.0 / 79.0
DHAP	169.0 / 97.0
D-Ribulose 5-phosphate	229.0 / 97.0
D-Xylulose 5-phosphate	229.0 / 139.0
Fumarate	115.0 / 71.0
Glyceraldehyde 3-phosphate	169.0 / 79.0
Lactate	89.0 / 45.0
Malate	133.0 / 115.0
NADH	666.0 / 649.0
NADP	742.0 / 620.0
NADPH	744.0 / 408.0
Nicotinamide adenine dinucleotide	662.0 / 540.0
Phosphoenolpyruvate	167.0 / 79.0
Pyruvate	87.0 / 32.0
Sedoheptulose 7-phosphate	289.0 / 79.0
Succinate	117.0 / 99.0
