# Supplementary material for: Granulocytic myeloid-derived suppressor cell activity during biofilm infection is regulated by a glycolysis/HIF1a axis
Source: J Clin Invest. 2024 Feb 29;134(8):e174051. doi: 10.1172/JCI174051 (PMC11014666; doi:10.1172/JCI174051)
Supplement: Unedited blot and gel images [file jci-134-174051-s013.pptx]

## Slide 1
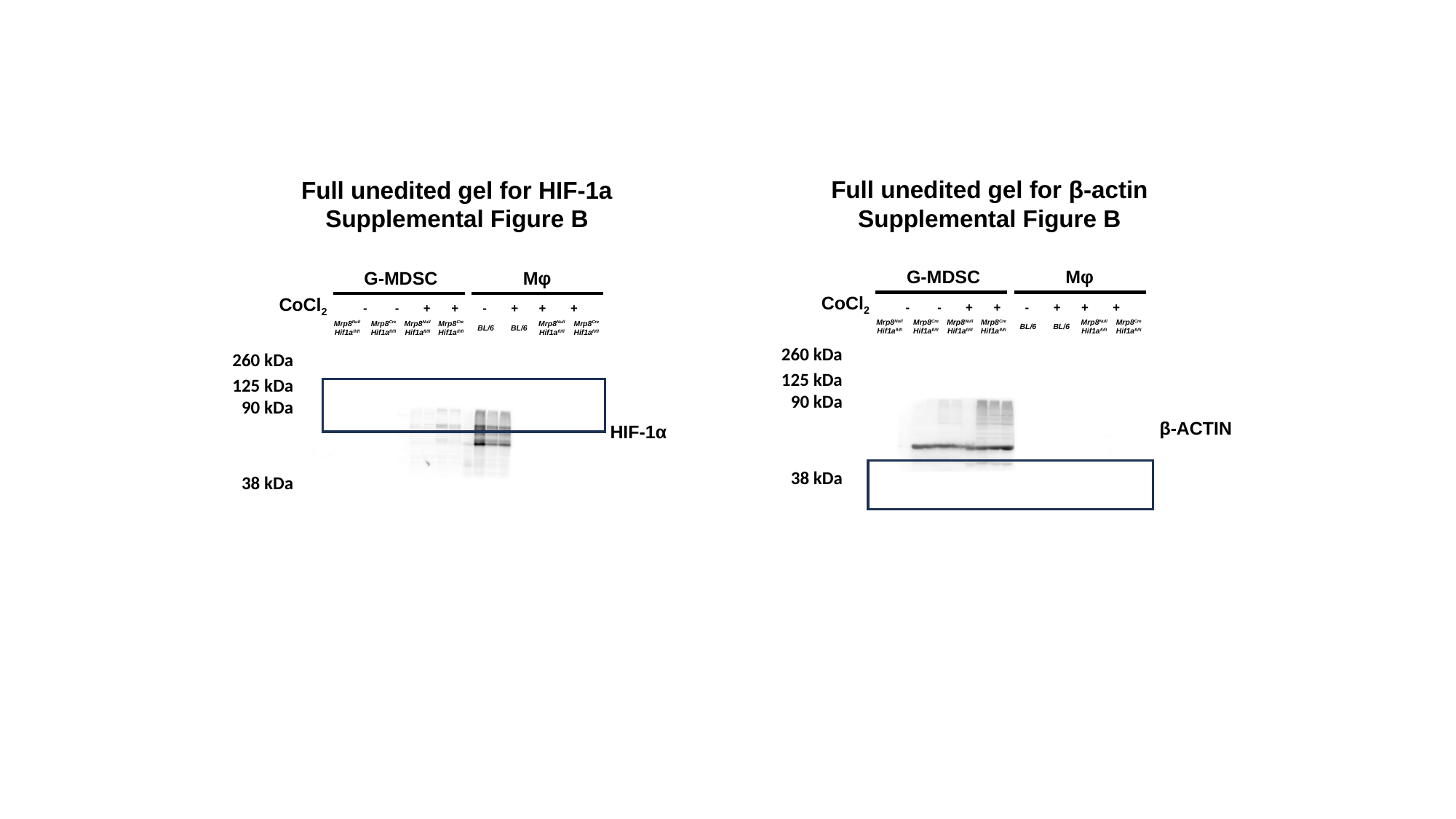

Full unedited gel for β-actin Supplemental Figure B
Full unedited gel for HIF-1a Supplemental Figure B
G-MDSC
Mφ
- - + + - + + +
CoCl2
Mrp8Null
Hif1afl/fl
Mrp8Cre
Hif1afl/fl
Mrp8Null
Hif1afl/fl
Mrp8Cre
Hif1afl/fl
Mrp8Null
Hif1afl/fl
Mrp8Cre
Hif1afl/fl
BL/6
BL/6
260 kDa
125 kDa
90 kDa
β-ACTIN
38 kDa
G-MDSC
Mφ
- - + + - + + +
CoCl2
Mrp8Null
Hif1afl/fl
Mrp8Cre
Hif1afl/fl
Mrp8Null
Hif1afl/fl
Mrp8Cre
Hif1afl/fl
Mrp8Null
Hif1afl/fl
Mrp8Cre
Hif1afl/fl
BL/6
BL/6
260 kDa
125 kDa
90 kDa
HIF-1α
38 kDa
